# MicroRNA-941 regulates the proliferation of breast cancer cells by altering histone H3 Ser 10 phosphorylation

**DOI:** 10.1038/s41598-020-74847-7

**Published:** 2020-10-21

**Authors:** Sunil Kumar Surapaneni, Zahid Rafiq Bhat, Kulbhushan Tikoo

**Affiliations:** Laboratory of Epigenetics and Diseases, Department of Pharmacology and Toxicology, National Institute of Pharmaceutical Education and Research (NIPER) S.A.S. Nagar, Sahibzada Ajit Singh Nagar, India

**Keywords:** Breast cancer, Epigenetics

## Abstract

Breast cancer including triple negative breast cancer (TNBC) represents an important clinical challenge, as these tumours often develop resistance to conventional chemotherapeutics. MicroRNAs play a crucial role in cell-cycle regulation, differentiation, apoptosis, and migration. Herein, we performed Affymetrix Gene Chip miRNA 4.0 microarray and observed differential regulation of miRNAs (75 upregulated and 199 downregulated) in metastatic MDA-MB-231 cells as compared to immortalized human non-tumorigenic breast epithelial (MCF-10A) cells. MicroRNA-941 was significantly upregulated in MDA-MB-231 cells (almost nine-fold increase) in comparison to MCF-10A cells. Transfection of MiRNA-941 inhibitor significantly decreased the proliferation and migration of MDA-MB-231 cells by altering the expressions of p21, Cyclin D1, PP2B-B1, E-cadherin and MMP-13. Interestingly, we provide first evidence that inhibiting miR-941 prevents cell proliferation and phosphorylation of histone H3 at Ser10 residue. Xenograft model of breast cancer was developed by subcutaneous injection of MDA-MB-231 cells into the mammary fat pad of female athymic nude mice (Crl:NU-Foxn1nu). The tumours were allowed to grow to around 60 mm^3^, thereafter which we divided the animals into seven groups (n = 5). Notably, intratumoral injection of miR-941 inhibitor significantly abolished the tumour growth in MDA-MB-231 xenograft model. 5-Fluorouracil (10 mg/kg, i.p.) was used as positive control in our study. To the best of our knowledge, we report for the first time that targeting miR-941 improves the sensitivity of MDA-MB-231 cells to 5-fluorouracil. This can be of profound clinical significance, as it provides novel therapeutic approach for treating variety of cancers (overexpressing miRNA-941) in general and breast cancers in particular.

## Introduction

Breast Cancer predominantly affects women in the United States and is the second underlying cause of cancer mortality among women. Currently, several management strategies of breast cancers are invasive with treatment associated side effects and there are limited effective therapeutic options available especially for triple negative breast cancer (TNBC)^1,2^. The majority of deaths in all cancers, including breast cancers result from tumours that regress by developing resistance to anti-cancer drugs^3,4^. Oncogenic targets, which are not inhibited by small molecule inhibitors can be targeted by application of RNA interference technology (i.e., small interfering RNAs/miRNAs) in cancer therapy^5,6^. MicroRNAs (miRNAs) are small (21–23 nucleotide) evolutionary conserved single stranded non-coding RNA molecules, which exert their effects by complementary base-pairing with 3′ untranslated regions (UTR) of target mRNAs. This causes either degradation or translational repression of the targeted miRNAs. MicroRNAs are involved in cell cycle progression, metabolism, cell death, angiogenesis, and metastasis by targeting oncogenes/tumour suppressor genes. MiRNA-based therapy involves either restoration of downregulated miRNAs or inhibition of over expressed miRNAs^7^. MiRNAs role in the initiation and progression of several human cancers, including breast cancer is well demonstrated^8,9^. MicroRNAs can sensitize tumour cells to various anticancer drugs. Anti-miR-21 sensitized breast tumour cells to topotecan (i.e., an inhibitor of DNA topoisomerase I), suggesting that suppression of oncogenic miR-21 sensitized breast tumour cells to topotecan^10^. On the other hand, liposomal delivery of tumour suppressor miRNA-34a decreased the proliferation and migration of breast tumours by down regulation of oncogenic genes (BCL-2 and SIRT1)^11,12^. MicroRNA expression profiles are used for the differentiation of tumour tissue from normal tissue, and prognosis of tumours^13^. MicroRNA expression profiling is used for the classification of several different human cancers (poorly differentiated tumour) and can also distinguish malignant tumours from benign tumours^14^. MiR-941 is reported earlier as an oncogene in melanoma and is also upregulated in non-small cell lung cancer^15,16^. DNA methylation regulates MiR-941 levels and 5-aza-2′-deoxycytidine (DNA methyltransferase inhibitor) induced overexpression of miRNA-941decreases the proliferation, migration and invasion of hepatoma, gastric cancer and colorectal cancer cells^17–19^. However, recent evidence revealed upregulation of miRNA-941 in hepatocellular carcinoma (HCC)^20^. MicroRNA-941 is also found to be upregulated and serves as promising diagnostic biomarker for ulcerative colitis and acute coronary syndrome^21,22^. Very few reports were found with relation to this miRNA in breast cancer^23,24^. To date, the biological effects of miRNA-941 in breast cancer cells are not yet explored.

## Material and methods

### Cell culture

MCF-10A, MDA-MB-231, MCF-7, A549 and A431 cells were acquired from American Type Cell Culture (ATCC, Manassas). PC-3 and DU-145 cells were purchased from NCCS Pune, India. BT-549 cell line was kindly gifted from Dr. Radha, Centre for Cellular and Molecular Biology, Hyderabad, India. Cells were grown under standard conditions in a controlled humidified atmosphere of 5% CO_2_ and 37 °C. These (A549, A431, MDA-MB-231, BT-549, MCF-7, DU-145 and PC3) cells were grown in Dulbecco’s modified eagle’s medium (DMEM) (Sigma Aldrich) supplemented with 10% fetal bovine serum (FBS) and 1% penicillin–streptomycin (GIBCO, U.S.A.). MCF-10A cells were grown in DMEM media supplemented with 10% horse serum, EGF (10 ng/mL), Hydrocortisone (500 ng/mL), Insulin (10 µg/mL), Cholera toxin (100 ng/mL) and 1% penicillin–streptomycin. Cell lines with passage number below 20 were used for our studies. Cells were routinely sub cultured using 0.25% trypsin/0.1% EDTA. All the other chemicals were procured from Sigma (St. Louis, MO, U.S.A.), unless otherwise mentioned.

### Total RNA isolation

Briefly, total RNA isolated from the cells using TRIzol reagent (Invitrogen, CA, USA) was purified using an RNeasy kit (AuPrep RNeasy Mini Kit; Life Technologies Pvt. Ltd., India). For validation of miRNAs through quantitative RT-PCR, we have isolated and purified total RNA by using miRCURY™ RNA isolation kit (Takara). The RNA quality and integrity of each sample were determined by measuring A260/280 absorbance ratio through Nanodrop spectrophotometer (ND-1000) and performing agarose gel electrophoresis.

### Affymetrix^®^ GeneChip^®^ miRNA Array

To identify the miRNAs involved in the progression of MDA-MB-231 cells, total RNA was isolated from MDA-MB-231 and MCF-10A (immortalized human non-tumorigenic breast epithelial) cells by using TRIzol reagent (Invitrogen) and was purified using an RNeasy kit (AuPrep RNeasy Mini Kit; Life Technologies Pvt. Ltd., India). These samples were further sent to iLife Discoveries for miRNA expression profiling. Herein, miRNA microarray was performed using Affymetrix Gene Chip miRNA 4.0 platform. Briefly, 2 μg of total RNA was tailed (by using 2.5 mM MnCl_2_, ATP, Poly A Polymerase-and incubation for 15 min at 37 °C), ligated (by using 1× Flash Tag ligation mix biotin, T4 DNA ligase and incubation for 30 min at room temperature) followed by addition of stop solution to stop the reaction. Each sample was then hybridized to a GeneChip^®^ miRNA v3Array (Affymetrix, Santa Clara, CA, USA) at conditions of 48 °C and 60 rpm for 16 h. Immediately following hybridization, automated washing and staining of hybridized probe arrays were carried out on Affymetrix GeneChip FS450 fluidic station and the arrays were finally scanned on the GeneChip^®^ Scanner 3000 7G Plus to detect the patterns of hybridization.

The hybridization data was collected as light emitted from incorporated fluorescent reporter groups of the targets upon binding to probe array. Probes that perfectly matched the target have produced stronger signals than those having mismatches. Data generated from the scan was then analyzed using the Affymetrix GeneChip^®^ Command Console software, and Affymetrix GeneChip^®^ Expression Console software. Metric analyses were carried out using the Affymetrix miRNA QC Tool software for quality control. The fold change values were summarized and normalized respectively using robust multi-array average (RMA) and variance stabilization (VSN) methods. GeneSpring GX software (Agilent Technologies) was used for bioinformatics analysis. Flow charts depicting Affymetrix Human miRNA profiling work flow and bioinformatics analysis of the data generated from Affymetrix 3000 7G scanner were shown in the Supplementary Figures S1 and S2.

### Reverse transcription and RT-PCR

For quality control and individual LNA RT-PCR assays, reverse transcription was carried out using the Universal cDNA Synthesis kit II (Exiqon) as described^25,26^. Real time PCR was performed at 95 °C for 10 min, followed by 40 cycles of 95 °C for 10 s/60 °C for 1 min using a Light Cycler 2.0 (Roche Diagnostics, USA). After amplification, a melt curve analysis was performed to assess the specificity of reaction. Relative miRNA expression was assessed through comparative Ct (ΔCt) method by considering the mean expression level of both SNORD49a and SNORA66 genes as a reference for comparison. For checking the mRNA levels of various genes, cDNA synthesis from RNA was carried out by using verso cDNA synthesis kit and then Quantitative RT-PCR was carried out as mentioned above. 18S rRNA was used as reference gene. The list of forward and reverse primer sequences of *PPP3R1*, *NDE1*, *RNF19A* and *GAB1* and 18S rRNA genes used for mRNA quantification through quantitative RT-PCR were given in Supplementary Table S1.

### Oligonucleotide transfection

MDA-MB-231 and MCF-10A cells seeded in 24 well plates (1 × 10^5^ cells per well) were allowed to grow in their respective media supplemented with 10% FBS and antibiotics (penicillin, 100 IU/ml and streptomycin, 100 μg/ml) under standard conditions in a controlled humidified atmosphere of 5% CO_2_ and 37 °C until they reached 70–80% confluency. Cells were incubated further in serum free media for 1 h and then transfected by MISSION^®^ Synthetic hsa-miR-941 inhibitor (HSTUD0972 from Sigma, St. Louis, MO, USA) and MISSION^®^ Synthetic negative control (NCSTUD002 from Sigma, St. Louis, MO, USA) respectively by using lipofectamine 3000 (Invitrogen, CA, USA) according to the manufacturer’s instructions. Negative control (cel-miR-243-3p) is a sequence from *Caenorhabditis elegans,* which has no homology to human and mouse gene sequences. Transfected cells were incubated for 24, 48, and 72 h depending on the studies being carried out. For western blotting and RT-PCR experiments, cells were seeded in 6 well plates (1 × 10^6^ cells per well) and then same experimental protocol was followed as described above.

### Cell viability assay

After transfection with MiR-941 inhibitor for respective time durations as described above, cell viability was assessed by using MTT assay as described^27^. The results were expressed either in absorbance, indicating the number of viable cells or by percentage cell viability. The viability of control cells was considered as 100%. Three independent experiments were performed for each study and all measurements were performed in triplicate.

### Monolayer wound healing assay

MDA-MB-231 cells were allowed to grow in 6-well plates supplemented with DMEM media to reach 70–80% confluency as described above. The cells were then incubated in reduced serum medium for 6 h containing 1% FBS. Cell cultures were scratched with a 200 µL sterile pipette tip and washed with PBS to remove detached cells and debris. Two crosses were scratched in each well, and the scratches were immediately subjected to photography using Nikon Inverted Microscope at 20 × magnification. Cells were then transfected with miR-941 inhibitor (50 nM) by using lipofectamine 3000. After 24 h, images of the same areas were acquired by using Nikon Inverted Microscope at 20 × magnification. The quantitative values of the percentage of cells covered in the scratch after 24 h were determined by using the web-based WimScratch module of Wimasis online software as described^28^. At least three biological replicates per experiment were used and the presented results were representative of triplicate experiments with similar outcome.

### Cell migration

MDA-MB-231 cells (5 × 10^4^ cells/well) were transiently transfected with miR-941 inhibitor (50 nM) by using lipofectamine 3000. After 24 h, cells suspended in 300 μL of serum free DMEM medium were seeded into the upper chamber of each insert (24-well insert; pore size, 8 μm; BD Biosciences). Afterwards, 500 μL of DMEM with 10% FBS was added into each wells of a 24-well plate. The inserts were incubated at 37 °C for 12 h in the wells containing DMEM media supplemented with serum. After incubation, migrated cells were washed thoroughly with DPBS, fixed (100% Methanol), followed by staining with crystal violet solution (0.5% crystal violet in 25% methanol/DPBS) for 15 min. Cells which did not migrate to the lower compartment of inserts were removed with a cotton swab. Photographs of each insert were taken in five random fields (40 ×). Quantification was expressed in terms of the percentage of area covered with migrated cells by using Image J software (National Institute of Health, USA).

### Chemosensitivity assay

MDA-MB-231 cells seeded in 24 well plates (1 × 10^5^ cells per well) were allowed to grow in DMEM medium as described above. Cells were incubated further in serum free media for 1 h and then transfected with MISSION^®^ Synthetic hsa-miR-941 inhibitor (HSTUD0972 from Sigma, St. Louis, MO, USA) and MISSION^®^ Synthetic negative control (NCSTUD002 from Sigma, St. Louis, MO, USA) using lipofectamine 3000 (Invitrogen, CA, USA) according to the manufacturer’s instructions. After 24 h, media was removed and PBS washing was carried out in miR-941 inhibitor group. Afterwards, these cells were treated with 5-Fluorouracil for another 24 h and finally cell viability was assessed.

### Isolation of total proteins and western blotting

Cytoplasmic and histone proteins were isolated and estimated according to the methods as described^27^. After isolation, equal amounts of protein were run on SDS PAGE and were electrophoretically transferred onto nitrocellulose membrane using semi-dry transfer apparatus (Bio-Rad) as described^26^. Immunoblot analysis was performed using anti-E-cadherin (rabbit, 1:500, Santa Cruz Biotechnology, CA, USA), anti-MMP-13 (rabbit, 1:1000, Santa Cruz Biotechnology, CA, USA), anti-CyclinD1 (rabbit, 1:500, Santa Cruz Biotechnology, CA, USA) anti-p21 (rabbit, 1:1000, Santa Cruz Biotechnology, CA, USA), anti-p-MSK-1 (Thr 581) (rabbit, 1:500,Cell signalling technology, USA), anti-MKP-1 (rabbit, 1:500, Santa Cruz Biotechnology, CA, USA), anti-PP2B-B1/2 (mouse, 1:1000, Santa Cruz Biotechnology, CA, USA), anti-histone H3 ser 10 phosphorylation (rabbit, 1:500, Cell signalling technology, USA), anti-H3 (goat, 1:1000 Sigma, St. Louis, MO, USA), anti-thymidylate synthetase (mouse, 1:1000, Santa Cruz Biotechnology, CA, USA)and anti α-tubulin (mouse, 1:1000, Santa Cruz Biotechnology, CA, USA) primary antibodies. The antigen-primary antibody complexes were incubated with horseradish peroxidase (HRP)-coupled secondary antibodies (Santa Cruz Biotechnology, CA, USA). Specific bands were detected and visualised according to the methods as described^26^. For subsequent antibody treatments, the membranes were stripped in stripping buffer and re-probed with another antibody. The immunoblots were quantified by densitometry scanning using NIH Image J software.

### In vivo tumour model

Animal protocol was approved by the Institutional Animal Ethics Committee (IAEC), NIPER (Protocol Approval No. IAEC 17/11). All experiments were performed as per the guidelines of the Committee for the Purpose of Control and Supervision of Experiments on Animals (CPCSEA), India and NIH guidelines (Guide for the care and use of laboratory animals). Female athymic nude mice (Crl:NU-Foxn1nu) were obtained from Vivo Bio Tech Ltd, Hyderabad and transferred to pathogen free environment of National Toxicology Center (NTC) in NIPER. The animals were maintained in sterile and clean cages with HEPA filters under standard diet with free access to water and controlled conditions of temperature: 20 ± 1 °C, humidity: 50 ± 10%; and 12 h light/dark cycle. All the animals were acclimatized for a period of one week prior to the start of experiments and were properly maintained by taking all necessary precautions and care.

#### Injection of MDA-MB-231 cells into nude mice

Xenografts of MDA-MB-231 cells were developed by subcutaneous injection of viable MDA-MB-231 (5 × 10^6^) cells dispersed in 1:1 (v/v) of PBS and matrigel into the fourth mammary fat pad on the right side of the nude mice. After 4 weeks when we have observed the animals have attained tumour volume of 60 mm^3^, we have randomly divided them into seven groups with each group consisting of five animals (n = 5). These seven groups include: cancer control, negative control, miR-941 inhibitor low dose (LD = 1 µM), miR-941 inhibitor medium dose (MD = 2 µM), 5-Fluorouracil (5-FU) as a positive control group, 5-FU + miR-941 inhibitor low dose (LD) and 5-FU + miR-941 inhibitor medium dose (MD). Animals were treated with miR-941 inhibitor (intratumoral), 5FU (i.p.) and combinations of miR-941 inhibitor and 5FU respectively for another four weeks before euthanization of mice. This was similar for all groups including the control group. The tumour volume was determined twice weekly during these 4 weeks’ treatments by measuring the tumour size with Vernier Caliper.

### Histopathological analysis of tumours

Tumour was removed from each animal, sliced and fixed in 10% v/v formal saline. Thereafter the tumours were subsequently embedded in paraffin; sections of 5 µm were prepared and mounted on slides previously coated with Mayer’s albumin. Sections were stained with hematoxylin & eosin to observe the structural changes as described^29^. Cover slip was mounted using DPX and observed at 20×, 40× magnification using OLYMPUS BX51 microscope and the images were captured with OLYMPUS DP 72 camera attached to the microscope^30^.

### Statistical analysis

All the values were expressed as mean ± S.E.M. Statistical comparison between two groups was done using t-test and comparison between more than two different groups was performed using one-way analysis of variance (ANOVA) followed by Tukey’s test. P value less than 0.05 was considered to be significant.

## Results

### MicroRNA expression signature

**Figure 1:**
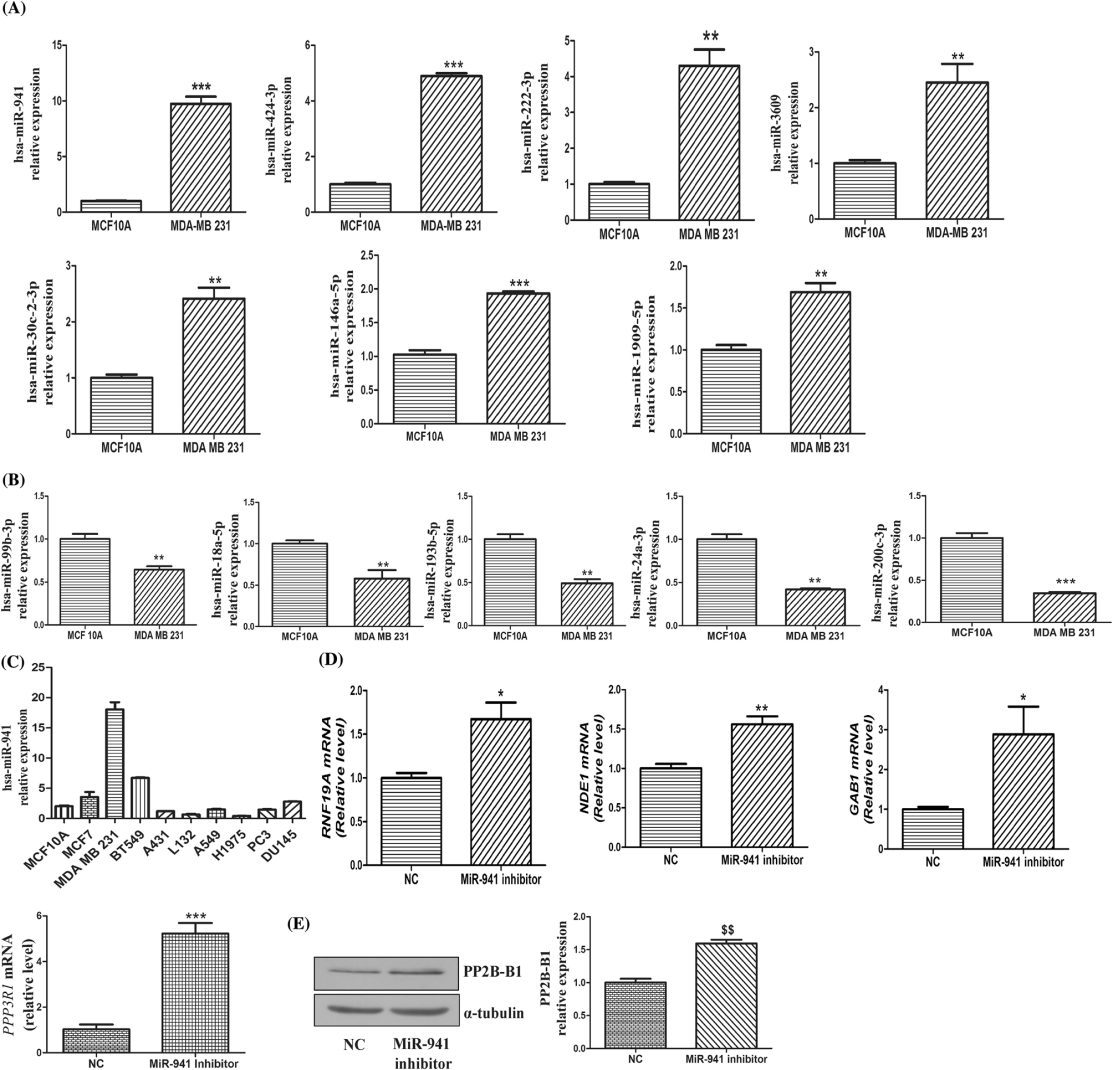
MicroRNAs expression in metastatic MDA-MB-231 cells. (**A**) Quantitative real-time PCR of upregulated miRNAs in MDA-MB-231 cell line when compared to MCF-10A cell line. (**B**) Quantitative real-time PCR of downregulated miRNAs in MDA-MB-231 cell line when compared to MCF-10A cell line. (**C**) Quantitative real-time PCR of relative expression of hsa-miR-941 in various cells. Results shown were representative of three different experiments. (**D**) Quantitative real-time PCR of genes like RNF19A, NDE1, GAB1 and PPP3R1 after transfection with MiR-941 inhibitor (50 nM) for 24 h in MDA-MB-231 cells. (**E**) Western blot and densitometric analysis of PP2B-B1 in MiR-941 inhibitor transfected MDA-MB-231 cells for 24 h. α-tubulin was used as loading control for our experiment. Results shown were representative of three different experiments. All values were expressed as mean ± S.E.M. *p < 0.05 **p < 0.01 ***p < 0.001 significant *vs* MCF-10A, negative control (NC); ^$$^p < 0.01 significant vs negative control (NC).

To identify the miRNAs involved in the growth and proliferation of breast cancer cells, miRNA microarray was performed using Affymetrix Gene Chip miRNA 4.0 platform. The number of differentially regulated miRNAs was determined by a fold change threshold of greater than or equal to 2 over the control sample (MCF-10A). Affymetrix Transcriptome Console analysis revealed that out of a total 266 differentially regulated miRNAs, 75 were upregulated and 191 were downregulated in MDA-MB-231 cells when compared to MCF-10A cells. Supplementary Figure S3 represents the scatter plot of differentially expressed miRNAs. Downregulated miRNAs constitute 71% of the total number of differentially regulated miRNAs. Hierarchical clustering analysis revealed the relative fold change increase or decrease of miRNAs expression respectively (Supplementary Figure S4; S4A upregulated and S4B downregulated). Tables S2 and S3 in the supplementary material lucidly show the upregulated and downregulated miRNAs in MDA-MB-231 cells when compared to MCF-10A cells, as shown in Supplementary Figures S4A and S4B. Differences in the fold change of various validated miRNAs between microarray and Quantitative RT-PCR techniques have been shown in Supplementary Table S4. Experimental validation of miRNA microarray data was performed by using quantitative RT-PCR for 14 randomly selected miRNAs (Seven upregulated and seven downregulated), based on their fold change values in the miRNA microarray data. A significant increase in the levels of miR-30c-2-3p, -424-3p, -941, -3609, -146a-5p, -1909-5p and -222-3p and significant downregulation in miR-193b-5p, -18a-5p, -24a-3p, -99b-3p and miR-200c-3p were found in MDA-MB-231 cell line when compared to MCF-10A cell line (Fig. 1A,B). However, we observed different patterns in the expression of hsa-miR-664b-5p and hsa-miR-1185-2-3p when validated through quantitative RT-PCR in comparison to miRNA microarray data (Supplementary Figure S5). The miRNA levels were normalized against SNORD49A and SNORA66 (reference genes). We next did literature survey to see if these validated differentially regulated miRNAs have been shown to be involved in cancers. Based on the literature search, we chose hsa-miR-941 for our further studies, as we observed significant upregulation (nine-fold higher) in MDA-MB-231 cells when compared to MCF-10A cells, and also there are no reports demonstrating the roles of miRNA-941 in breast cancers including TNBC. Next, we checked the relative expression levels of hsa-miR-941in various cancer cell lines such as lung (A549), prostate (PC3, DU-145), breast (MCF-7, MDA-MB-231 and BT-549) and skin (A431) by performing quantitative RT-qPCR (Fig. 1C). MiR-941 was found to be highly upregulated in human breast cancer cells (MDA-MB-231, BT-549 and MCF7) cell lines when compared to other cancer cell lines. MiR-941 was found to be 1.75, 3 and nine-fold higher in MCF7, BT-549 and MDA-MB-231 cells respectively when compared to MCF-10A cells. Apart from miRNAs, a number of small nucleolar RNAs (snoRNAs) were also found to be differentially regulated. Out of the 80 downregulated snoRNAs, 51 were C/D box and 20 were H/ACA box type. C/D box snoRNAs are known to be involved in rRNA complementarity and site specific ribosome methylation. H/ACA box snoRNAs facilitate the conversion of uracil to pseudouracil. The rest of snoRNAs can either belong to the RNA component of RNase MRP (Mitochondrial RNA Processing endoribonuclease) or may be involved in tRNA processing. The three upregulated snoRNAs in our study may belong to the third group of snoRNAs. Differentially expressed snoRNAs were shown in Supplementary Table S5.

### MiRNA-941 inhibitor altered the expression of *RNF19A, NDE1, GAB1* and *PPP3R1*genes

To determine the potential target genes of miR-941, miRSystem tool (https://mirsystem.cgm.ntu.edu.tw/) was used. MiRSystem is a database, which uses experimentally validated data from TarBase and miRecords. It also uses seven well known target prediction databases (i.e., PITA, miRanda, DIANA-microT, mirBridge, PicTar, rna22, TargetScan) and five pathway databases (i.e., Gene Ontology, KEGG, BioCarta, Pathway Interaction Database and Reactome) to predict the target genes of miRNAs and determine the biological functions and various signalling pathways of the target genes respectively^31^. When the query (hsa-miR-941) was submitted in miRSystem, it has generated the target gene summary report, functional annotation summary report and pathway ranking summary. MiRSystem analysis revealed that miR-941 regulates many genes, which are involved in cell proliferation, apoptosis, inflammation, autophagy and cell cycle arrest. Out of these, top 15 miRSystem predicted targets of miR-941 were selected for validation through quantitative RT-PCR to check alterations in their gene expression by inhibition of miRNA-941 in MDA-MB-231 cells. MiRNA-941 inhibitor significantly upregulated the mRNA levels of genes like *RNF19A*, *NDE1*, *GAB1* and *PPP3R1* when compared to negative control (Fig. 1D). *NDE1* gene encodes a member of the nuclear distribution E (NudE) family of proteins, whose levels vary depending upon the cell cycle stage i.e., its levels are reduced when cells enter G1 and increased when cells are in mitosis^32^. *RNF19A* encodes a member of the ring between ring fingers (RBR) protein family. RNF19A, an E3 ubiquitin ligase plays a crucial role in inhibition of NF-κB signalling through the degradation of TRAF6^33^. The protein encoded by *GAB1* gene is a member of the IRS1-like multisubstrate docking protein family. Gab1 plays an important role in tumorigenesis by interacting with c-Met receptor signalling^34^. PP2B-B1 is a protein phosphatase involved in the dephosphorylation of various proteins on serine and threonine residues, thereby regulating cell division, homeostasis and apoptosis^35^. Among the various validated miR-941 targets through quantitative RT-PCR, we have seen maximal change in the *PPP3R1* gene (i.e., five-fold higher) after transfection with miR-941 inhibitor. So, we have checked the expression of PP2B-B1 at the protein level. We observed a significant increase in its expression with miR-941 inhibitor when compared to negative control in MDA-MB-231 cells (Fig. 1E).

### Inhibition of microRNA-941 decreased the proliferation of MDA-MB-231 cells

**Figure 2:**
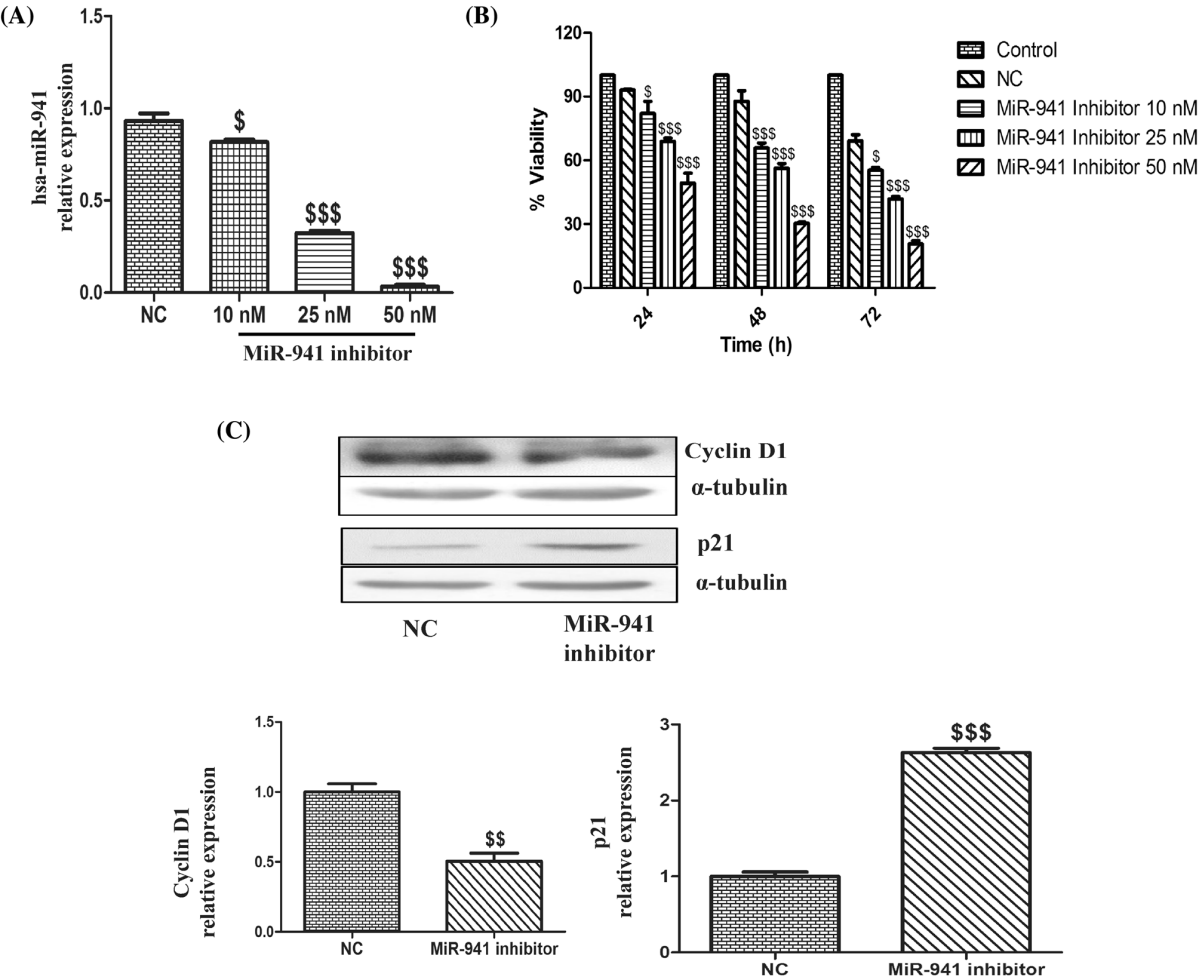
Inhibition of microRNA-941 decreased the proliferation of MDA-MB-231 cells. (**A**) Quantitative real-time PCR of hsa-miR-941 after transfected with MiR-941 inhibitor for 24 h in MDA-MB-231 cells. (**B**) MTT assay of MiR-941 inhibitor on the proliferation of MDA-MB-231 cells. (**C**) Western blots and densitometric analysis of p21 and Cyclin D1 in MiR-941 inhibitor transfected MDA-MB-231 cells for 24 h. Three independent experiments were performed for each cell type. All values expressed as mean ± S.E.M. ^$^p < 0.05, ^$$^p < 0.01, ^$$$^p < 0.001 significant vs negative control (NC).

RT-qPCR data revealed that MiR-941 inhibitor significantly decreased miRNA-941 levels in a dose-dependent manner in MDA-MB-231 cells (Fig. 2A). To characterize the effects of miR-941 on cell growth, MDA-MB-231 cells were transfected with MiR-941 inhibitor at different doses of 10 nM, 25 nM and 50 nM for a period of 24, 48 and 72 h. A significant dose-dependent decrease in the growth of cells was observed in comparison to negative control (NC), which further confirms that decreased proliferation of MDA-MB-231 cells by miRNA-941 inhibitor was due to inhibition of miRNA-941. MDA-MB-231 cells transfected with miRNA-941 inhibitor at 50 nM showed 51%, 70% and 80% decrease in cell growth for 24, 48 and 72 h, respectively (Fig. 2B). To further explore the molecular mechanism underlying the biological effects of miR-941 inhibitor-induced growth inhibition in MDA-MB-231 cells, the protein expressions of various cell cycle regulator proteins (p21 and Cyclin D1) were checked. A significant decrease in the protein expression of Cyclin D1 levels and a significant increase in p21 expression levels were observed in MiR-941 inhibitor transfected cells when compared to NC (Fig. 2C).

### MicroRNA-941 inhibitor impeded the migration and invasion of MDA-MB-231 cells

**Figure 3:**
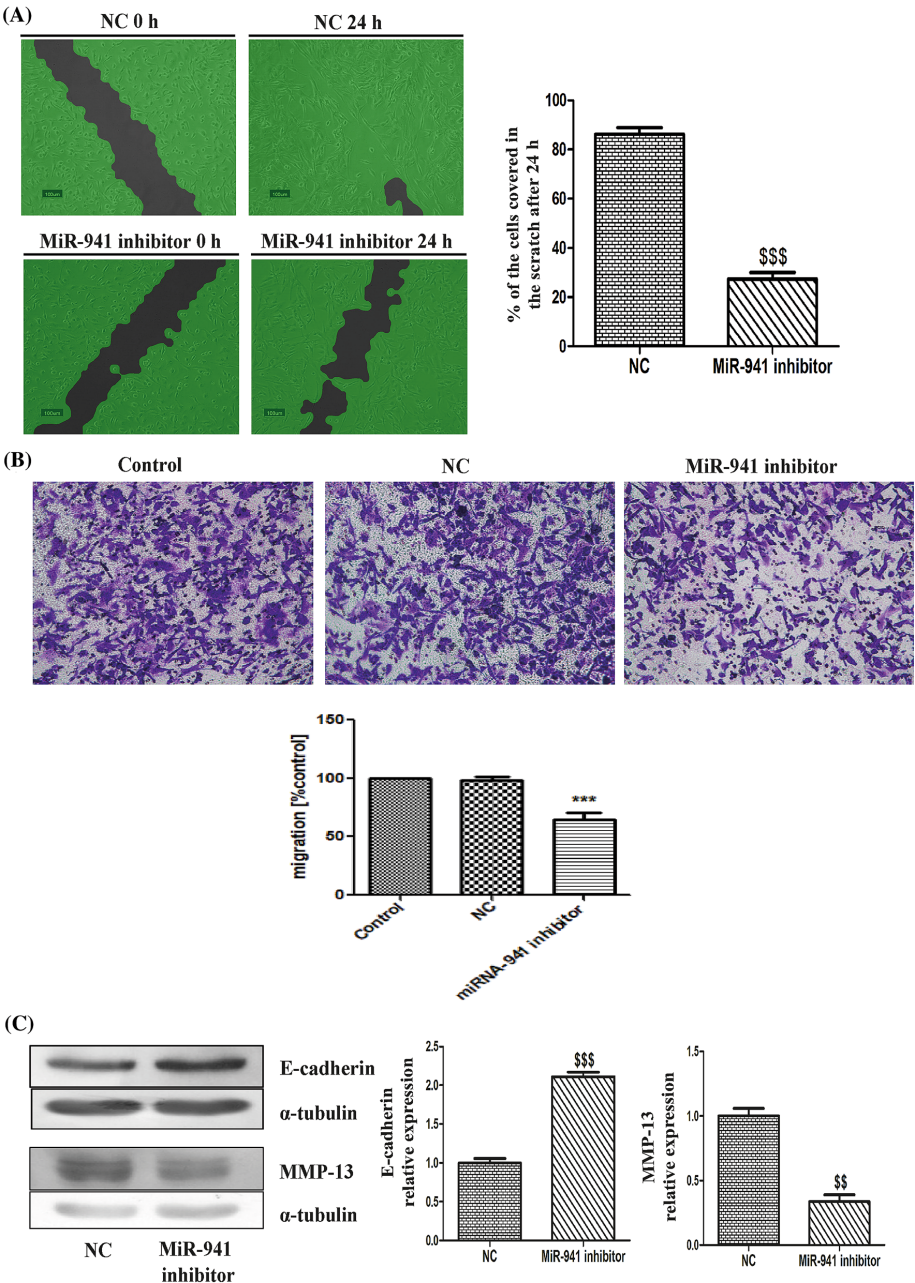
MicroRNA-941 inhibitor impeded the migration and invasion of MDA-MB-231 cells. (**A**) Scratch wound images and WimScratch tool quantitative analysis of percentage of the cells covered in the scratch after 24 h transfection with miRNA-941 inhibitor (50 nM) in comparison to negative control. (**B**) Transwell migration assay with miR-941 inhibitor in MDA-MB-231 cells. (**C**) Western blotting and densitometric analysis of E-cadherin and MMP-13 in MiR-941 inhibitor (50 nM) transfected MDA-MB-231 cells for 24 h. α-tubulin was used as loading control for our experiment. Results shown were representative of three different experiments. All values were expressed as mean ± S.E.M. ^$$^p < 0.01, ^$$$^p < 0.001 significant vs negative control (NC).

To evaluate the impact of microRNA-941 on the migration of MDA-MB-231 cells, wound healing assay was performed. Interestingly, miR-941 inhibitor significantly inhibited tumour cell migration when compared to negative control (Fig. 3A). WimScratch tool quantitative analysis revealed that 86% of the scratch area was covered with cells in negative control group and 28% of the scratch area was covered with cells in miRNA-941 inhibitor transfected group (Fig. 3A). Moreover, transwell migration assay results revealed that miR-941 inhibitor significantly decreased the migration (i.e., decreased the number of cells to migrate to the lower surface of the membrane) of MDA-MB-231 cells in comparison to negative control (Fig. 3B). To further confirm whether miR-941 is involved in the migration and invasion of MDA-MB-231 cells, the protein expressions of E-cadherin (Epithelial cell marker) and MMP-13 (tumour marker for invasive breast cancer) were checked. MiR-941 inhibitor significantly increased E-cadherin and decreased MMP-13 protein expression respectively in MDA-MB-231 cells. This suggests that miRNA-941 inhibitor decreases the migration and invasion of MDA-MB-231 cells (Fig. 3C).

### MicroRNA-941 inhibitor decreased the expression of Histone H3 ser10 phosphorylation in MDA-MB-231 cells

**Figure 4:**
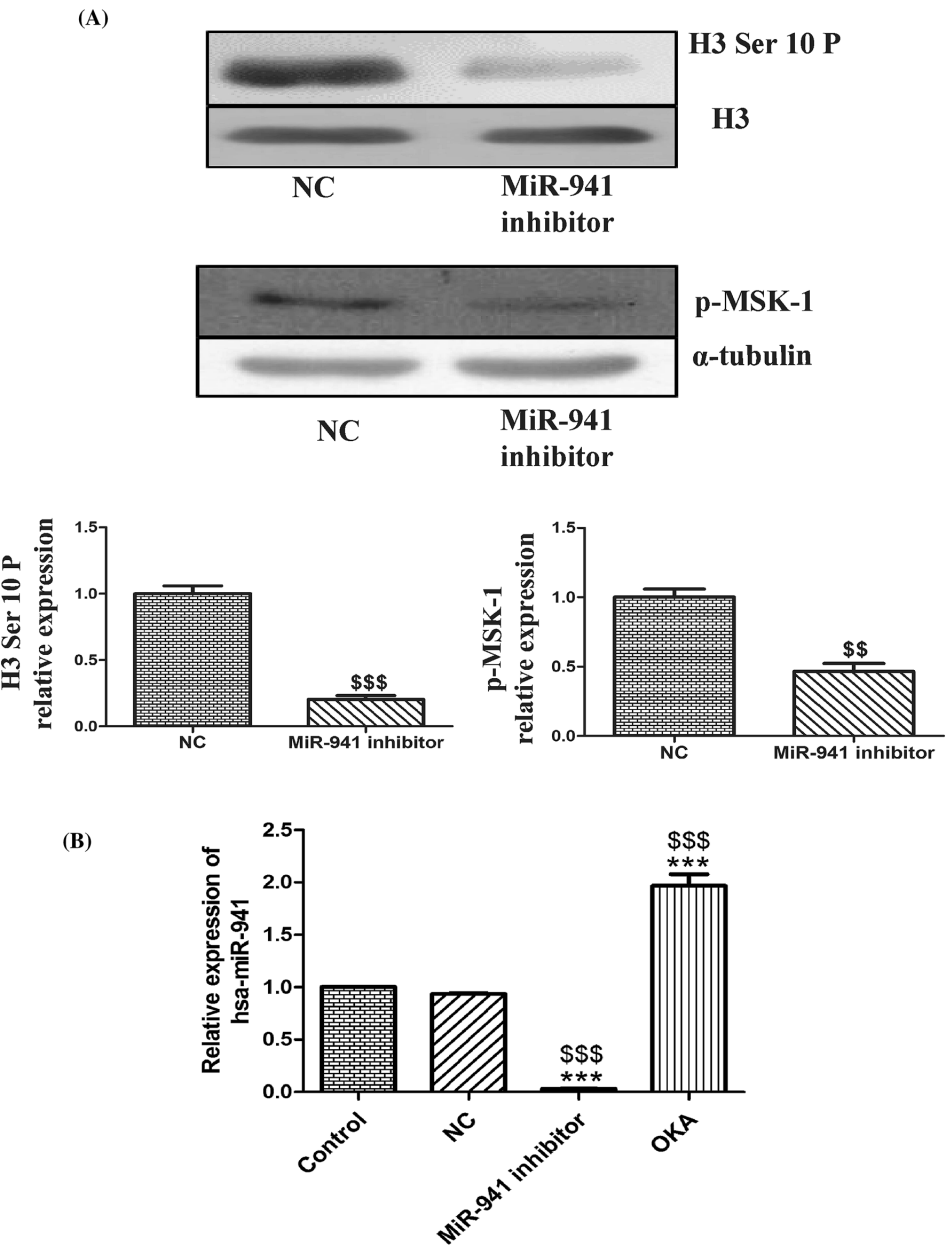
Epigenetic alterations of microRNA-941 inhibitor in MDA-MB-231 cells. (**A**) Western blots and densitometric analysis of p-H3 (Ser 10) and p-MSK-1 in MiR-941 inhibitor transfected MDA-MB-231 cells for 24 h. (**B**) Quantitative real-time PCR based relative expression of hsa-miR-941 in MDA-MB-231 control, negative control, and miRNA-941 inhibitor and okadaic acid (5 nM) treatment group. α-Tubulin and H3 were used as loading controls for our experiment. Three independent experiments were performed for each cell type. All values were expressed as mean ± S.E.M. ***p < 0.001 significant vs untreated MDA-MB-231 cells (control group). ^$$^p < 0.01, ^$$$^p < 0.001 significant vs Negative control (NC).

Histone modifications play a crucial role in cell cycle regulation, and phosphorylation of histone H3 is well known to be associated with chromatin condensation prior to mitosis^36,37^. As miR-941 inhibitor significantly altered the expression of cell cycle regulatory genes (Cyclin D1 and p21), we want to check the involvement of histone H3 serine 10 phosphorylation in miR-941 inhibitor induced-cell cycle alterations. MiR-941 inhibitor significantly decreased histone H3 serine 10 phosphorylation in MDA-MB-231 cells when compared to negative control. Further, we also checked the expression of proteins (i.e., p-MSK-1 and MKP-1) for determining the possible factors responsible for decreased histone H3 serine 10 phosphorylation. A significant decrease in the protein expression of p-MSK-1 was observed withmiR-941 inhibitor in MDA-MB-231 cells (Fig. 4A). We did not observe any alterations in the expression of MKP-1 with miR-941 inhibitor. Since MiR-941 inhibitor significantly decreased the histone H3 Ser10 phosphorylation, we want to check whether treatment by okadaic acid (5 nM; a phosphatase inhibitor) leads to the alteration of miRNA-941 levels in MDA-MB-231 cells. Interestingly, okadaic acid (OKA) treatment alone increased the levels of hsa-miR-941 in MDA-MB-231 cells (Fig. 4B). This suggests that miR-941 might regulate the proliferation of MDA-MB-231 cells by altering histone H3 ser10 phosphorylation.

### MicroRNA-941 inhibitor increased the sensitivity of 5-fluorouracil in MDA-MB-231 cells

**Figure 5:**
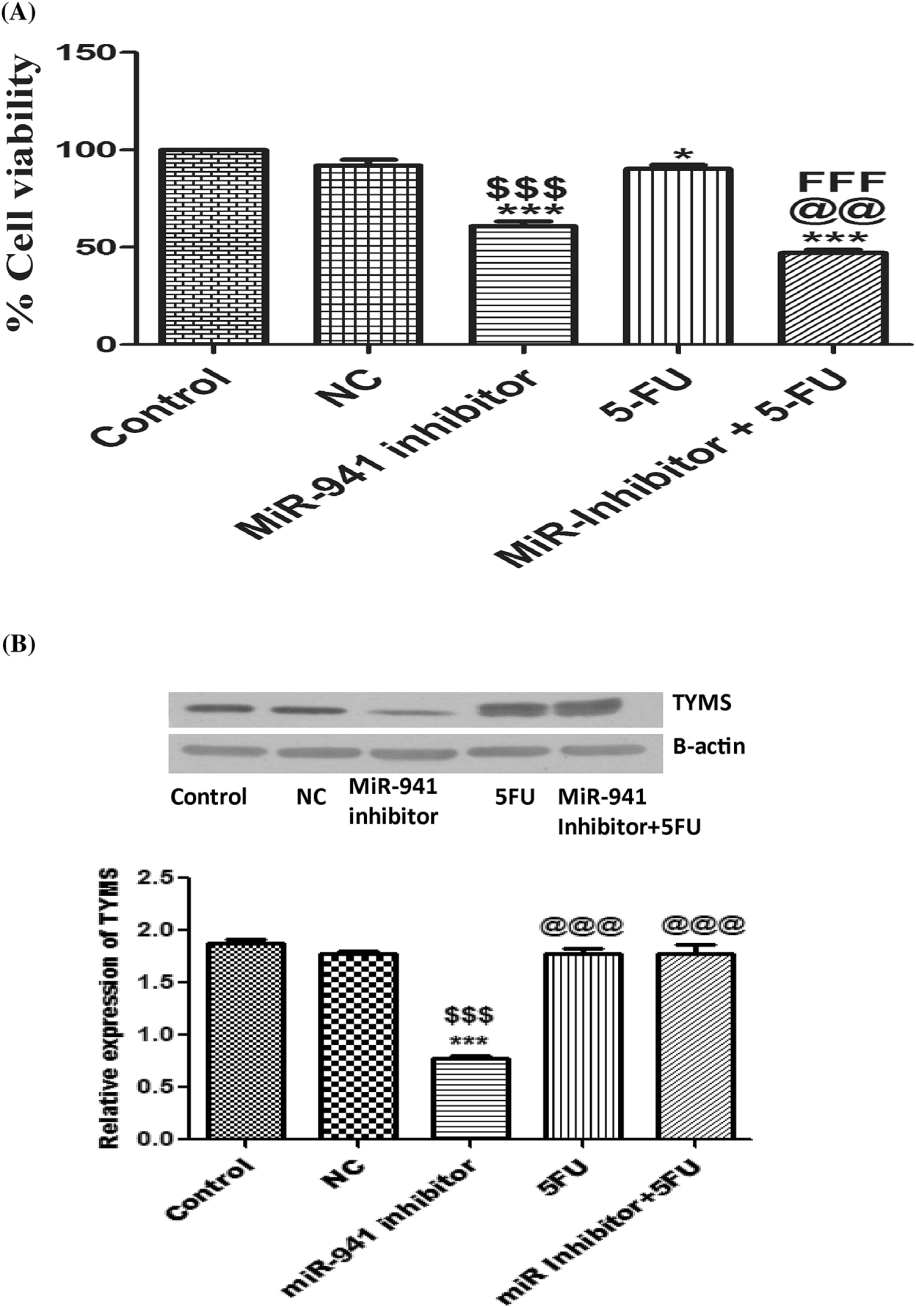
MicroRNA-941 inhibitor increased the sensitivity of MDA-MB-231 cells to 5-Fluorouracil. (**A**) MTT assay of pre-treatment with MiR-941 inhibitor for 24 h and after that, the cells of the respective groups were treated with 5-Fluorouracil for another 24 h. (**B**) Western blots and densitometric analysis of the protein expression of thymidylate synthetase (TS) in miR-941 inhibitor transfected MDA-MB-231 cells for 24 h, 5FU alone group and pre-treated miR-941 inhibitor followed by 5FU post treatment group. β-Actin was used as loading control for our experiment. Three independent experiments were performed for each cell type. All values were expressed as mean ± S.E.M. *p < 0.05, **p < 0.01, ***p < 0.001 significant vs untreated MDA-MB-231 cells (control group). ^$$^p < 0.01, ^$$$^p < 0.001 significant vs Negative control (NC); ^@@^p < 0.01, ^@@@^p < 0.01 significant vs MiRNA-941 inhibitor; ^FFF^p < 0.001 significant vs 5-Fluorouracil.

MicroRNA-941 inhibitor significantly decreased the proliferation of MDA-MB-231 cells. MicroRNAs can modulate the sensitivity of various chemotherapeutic agents in cancer cells^38,39^. Herein, we checked whether miRNA-941 inhibitor can increase the sensitivity of 5-fluorouracil (5-FU) in MDA-MB-231 cells. 5-FU decreases the proliferation of MDA-MB-231 cells in a dose-dependent manner and its IC_50_ value was observed to be 48.7 ± 2.3 µM after 96 h treatment^40^. Recently, we have reported that 5-FU induces only 12% cell death at 75 μM in MDA-MB-231 cells^27^. Herein, 25 μM concentration of 5-FU was used for checking whether miRNA-941 inhibitor (50 nM) pre-treatment for 24 h could sensitize the MDA-MB-231 cells to 5-FU. Interestingly, we observed that pre-treatment with miRNA-941 inhibitor increased the sensitivity of 5-FU around 44% more when compared to 5-FU alone in MDA-MB-231 cells (Fig. 5A). 5-FU induced only 13% death after 24 h in MDA-MB-231 cells. Moreover, pre-treatment with miRNA-941 inhibitor increased the sensitivity of 5-FU around 13% more in comparison to MiR-941 inhibitor alone. Reports suggest that cancer cells show resistance to 5-FU due to increased expression of Bcl-2, BCL-XL and thymidylate synthetase^41,42^. Herein, we observed that miR-941 inhibitor significantly decreased the protein expression of thymidylate synthetase in MDA-MB-231 cells (Fig. 5B). MiRNA-941 inhibitor pretreatment did not significantly reduce the expression of thymidylate synthetase after post treatment with 5-FU, suggesting that alterations in cell cycle proteins such as p21 and CyclinD1 might be responsible for improved sensitization of 5-FU in MDA-MB-231 cells.

### MicroRNA-941 inhibitor exerted anti-cancer effect in MDA-MB-231 xenografts

**Figure 6:**
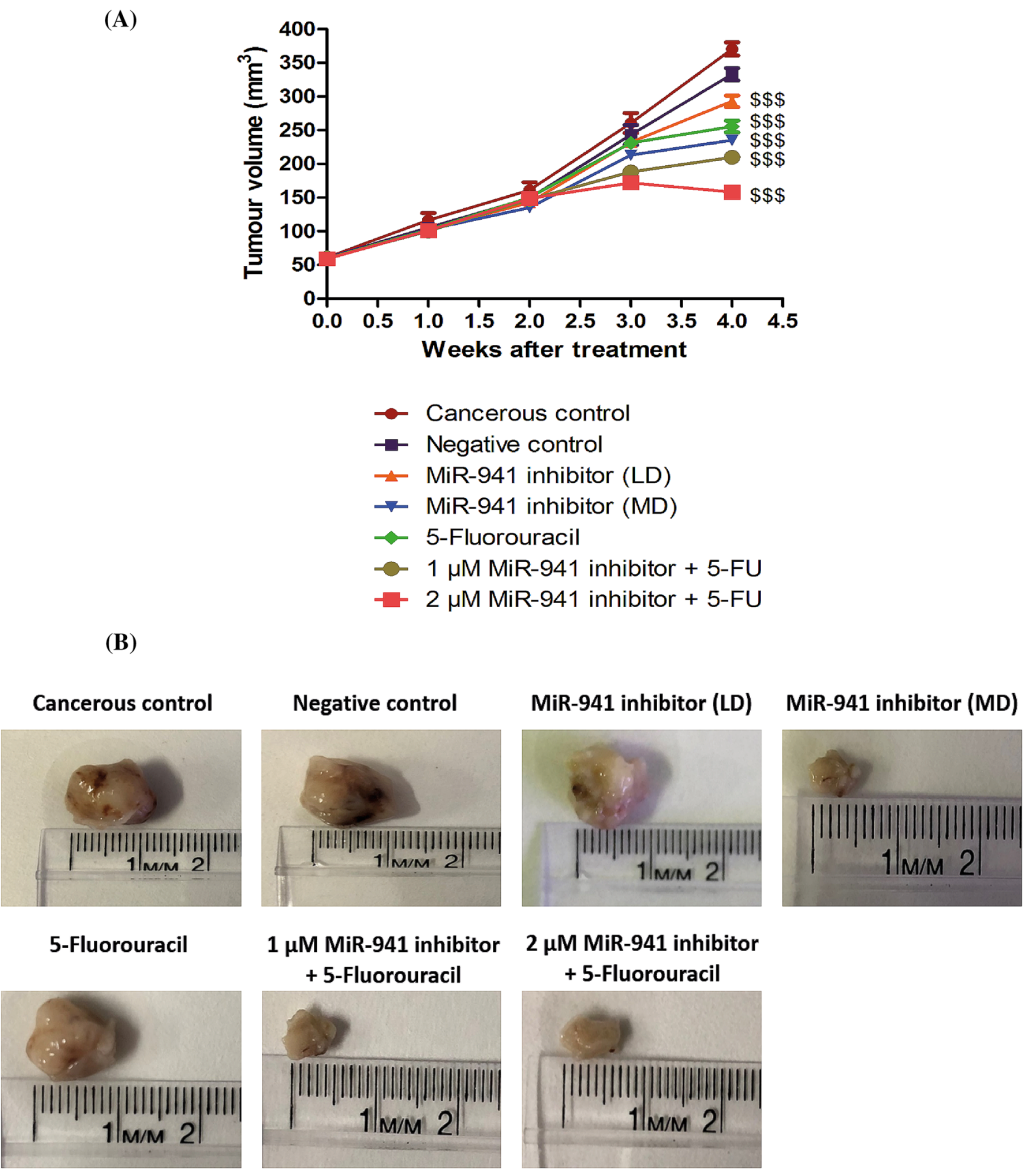
MicroRNA-941 inhibitor showed anti-cancer effect in MDA-MB-231 xenograft model of nude mice. (**A**) Comparative anti-cancer activity in MDA-MB-231 subcutaneous xenograft nude mice model of triple negative breast cancer. Triple negative breast cancer was induced in nude mice by implanting MDA-MB-231 triple negative breast cancer cells into the mammary fat pad of the mice. The tumour was allowed to grow till 60 mm^3^. Thereafter, treatments were evaluated over a period of 4 weeks. Data represents mean ± S.E.M. of 5 mice per group. ^$$$^p < 0.001 significant vs negative control. (**B**) Representative tumours excised out of mice at end point for untreated (Cancerous control group), negative control (scrambled miRNA sequence), Low dose MiR-941 inhibitor (LD = 1 µM), Medium dose MiR-941 inhibitor (MD = 2 µM), Positive control 5-Fluorouracil (25 µM), Low dose 1 µM MiR-941 inhibitor + 5-FU (25 µM) and 2 µM MiR-941 inhibitor + 5-FU (25 µM) treated mice after a period of 4 weeks as described in “Material and methods”.

**Figure 7:**
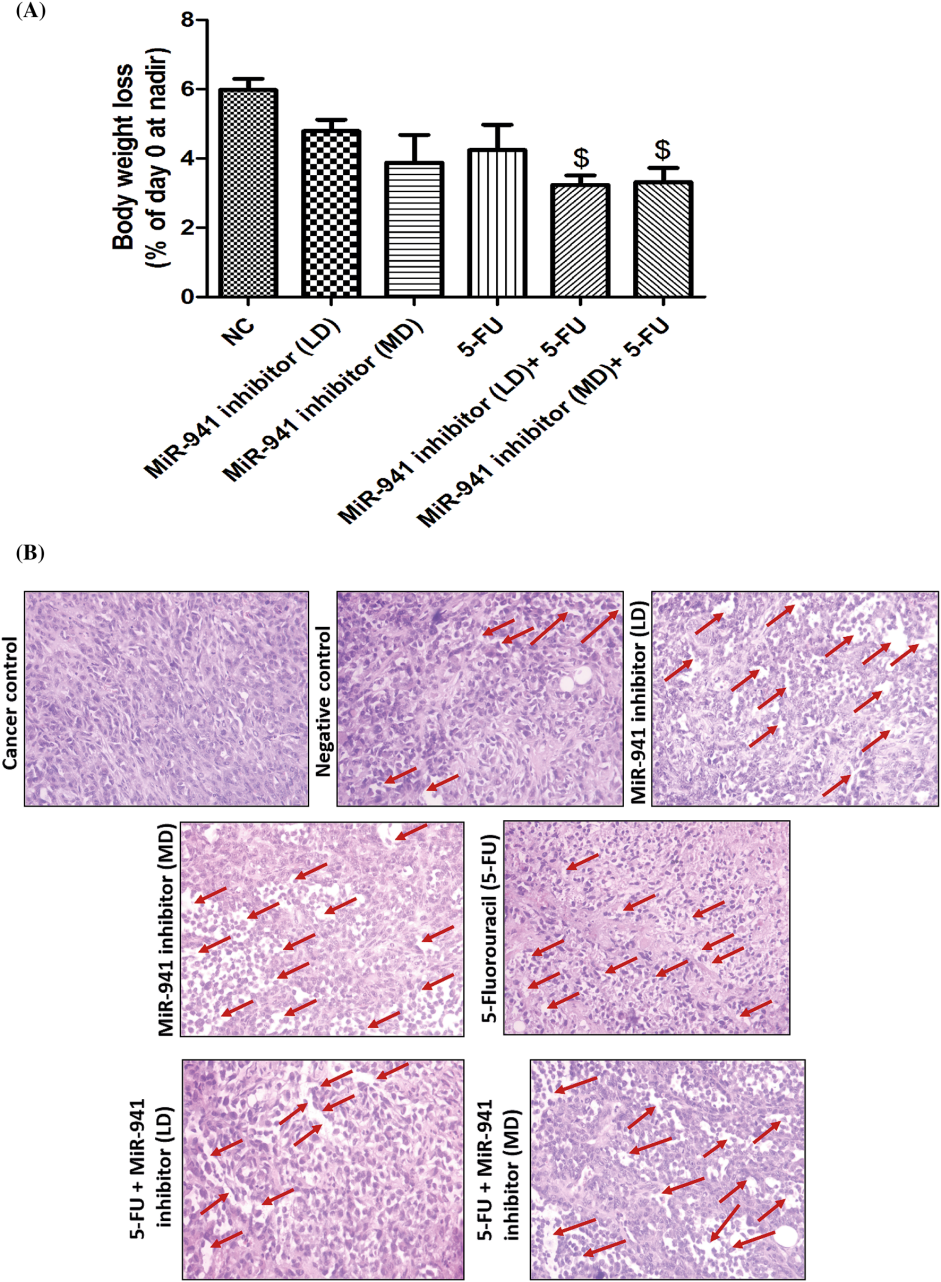
MiRNA-941 inhibitor and 5-FU combination exhibit high efficacy and low toxic effects in MDA-MB-231 xenograft model of nude mice. (**A**) Body weight loss of animals after dosing of animals: BWL was calculated after 4 weeks of treatment. (**B**) Histopathological analysis of tumours excised from (a) cancer control (b) negative control (scrambled miRNA sequence) (c) MiR-941 inhibitor (LD = 1 µM), (d) MiR-941 inhibitor (MD = 2 µM), (e) 25 µM 5-fluorouracil (5-FU), (f) 5-fluorouracil + MiR-941 inhibitor (LD) and (g) 5-fluorouracil + MiR-941 inhibitor (MD) treated animals. The arrows depict the interstitial spaces in tumour samples. No such significant spaces were found in tumours of untreated animals. All values were expressed as mean ± S.E.M. ^$^p < 0.05 significant vs Negative control (NC).

We observed significant anticancer activity of microRNA-941 inhibitor in MDA-MB-231 cells (in-vitro). Further, evaluation of antitumor efficacy of miR-941 inhibitor in vivo requires the development of a xenograft animal model for breast cancer using MDA-MB-231 cells. In order to ensure whether miR-941 inhibitor also shows good anti-cancer effects in vivo, we developed xenograft model of triple negative breast cancer, by subcutaneous injection of MDA-MB-231 cells into the mammary fat pad of nude mice and evaluated its (miR-941 inhibitor) efficacy. Tumours were allowed to grow to around 60 mm^3^ tumour volume and thereafter, the animals were divided into seven groups (n = 5). Control mice received 0.9% saline solution. The tumour volume was checked twice weekly up to four weeks. MicroRNA-941 inhibitor (2 µM) treatment significantly led to suppression of MDA-MB-231 tumour volume in comparison to control groups, suggesting that miR-941 inhibitor could inhibit tumour growth in vivo (Fig. 6A). 5-fluorouracil (5-FU) was used as positive control in our study. 5-FU alone caused suppression of tumour volume to some extent. MiR-941 inhibitor also significantly sensitized the tumour cells to 5-FU in-vivo and thereby caused increased suppression of tumour volume (Fig. 6A). MiR-941 inhibitor significantly decreased the size of the tumours when compared to negative control group. MiR-941 inhibitor also increased the sensitivity of 5-FU in vivo, as evidenced by its decreased tumour size in comparison to 5-FU alone group (Fig. 6B). Body weight loss (BWL) was assessed in all animals of each group to determine the toxicological effects of each treatment. Control animals showed body weight loss coincided with the tumour load. After 4 weeks, miR-941 inhibitor, 5-fluorouracil and combination groups of miR-941 inhibitor with 5-fluorouracil treated animals showed around 3.2–4.9% loss in body weight (Fig. 7A). Mice were sacrificed and the excised tumours were used for histopathology to determine their structural/morphological changes with each treatment. Significant cytotoxic damage was observed in the tumour tissues of miR-941 inhibitor, and miR-941 inhibitor + 5-fluorouracil treated animals. 5-FU showed some degree of cytotoxicity to the tumours. We did not observe any significant damage in the tumours of cancer control and negative control groups (Fig. 7B). The histopathological (H&E) staining of the tissues clearly indicates an increase of interstitial spaces in the tumours of miR-941 inhibitor, and miR-941 inhibitor + 5-fluorouracil treated animals. The enhanced sensitivity of 5FU in miR-941 inhibitor + 5-fluorouracil group might be due to increased penetration of 5FU to the tumours due to increased interstitial spaces by miR-941 inhibitor.

## Discussion

As microRNAs are differentially expressed in normal and tumour tissues, multiple studies have demonstrated their potential in tumour profiling, cancer diagnosis and prognosis^43,44^. In our study, miRNA microarray was performed for both MDA-MB-231 (tumorigenic human breast cancer cell line) and MCF-10A (non-tumorigenic human breast epithelial cell line) by using Affymetrix miRNA 4.0 platform. Differential analysis by Affymetrix Transcriptome Console software revealed upregulation of 71 mature miRNAs and downregulation of 112 mature miRNAs in MDA-MB-231 cells when compared to their (mature miRNAs) levels in MCF-10A cells. Moreover, some snoRNAs and stem loop miRNAs were differentially regulated in MDA-MB-231 cells when compared to their levels in MCF-10A cells. Quantitative real-time PCR was performed for validation of 14 randomly chosen miRNAs (seven upregulated and seven downregulated). We observed increased levels of miR-424-3p, -146a-5p, -1909-5p, -941, -222-3p, -30c-2-3p and -3609 while decreased levels of miR-200c-3p, -18a-5p, -24-3p, -99b-3p, and -193b-5p in MDA-MB-231 cells. However, we observed different trend in the levels of miR-664b-5p and -1185–2-3p during RT-qPCR validation of microarray data. These discrepancies could be due to different probe designs, hybridization conditions, labelling chemistries and differences in the abundances of miRNA in the samples^45^. Previous reports demonstrate that abundant miRNAs showing even twofold change in their levels can significantly alter the mRNA of the proteins. Moreover, less abundant miRNAs can be used as specific biomarkers for various diseases^46^. It was observed that miR-941 levels were higher (i.e., around nine fold) in MDA-MB-231 cells when compared to those in MCF-10A cells. MiR-941 levels were also higher in MDA-MB-231 cells in comparison to MCF-10A, MCF-7, BT-549, L132, A431, A549, PC3 and DU-145 cells.

Currently there are many tools for predicting miRNA targets but each tool uses different algorithms, assigns different ids to the miRNAs and gives score to the targets differently. This makes it difficult to predict and analyse the biological pathways altered by target genes. MiRSystem is a database, which uses seven algorithms and five pathway databases for the simultaneous prediction of miRNA targets and the biological pathways altered by target genes^31^. MiRSystem analysis revealed various targets for miR-941 (i.e. *SUV420H2*, *PTPRS*, *ST5*, *GAB1*, *KCMF1*, *NDE1*, *GRB2*, and *PPP3R1* etc.). Validation of miRNA-941 targets was performed by quantitative RT-PCR after transfecting MDA-MB-231 cells with miR-941 inhibitor. MicroRNA-941 inhibitor significantly increased (around five-fold) the expression of *PPP3R1* (formerly known as *PP2B-B1*) gene, which is one of the predicted miR-941 targets by miRSystem. MiR-941 inhibitor transfected MDA-MB-231 cells showed a significant increase in the expression of PP2B-B1 protein, which further confirms it as the target of miR-941. PP2B-B1, a protein phosphatase is involved in the dephosphorylation of various proteins on serine and threonine residues, thereby regulating cell division, homeostasis and apoptosis^35^. Some studies have shown that PP2B activation is responsible for decreased cyclin levels^47,48^.

MiR-941 has been reported as an oncogene in melanoma and non-small cell lung cancer^15,16^. We observed that miR-941 inhibitor significantly decreased the proliferation of MDA-MB-231 cells in a dose- and time-dependent manner, which coincided with a significant decrease in miR-941 levels in a dose-dependent manner when compared to negative control.

Since miR-941 inhibitor significantly decreased the proliferation of MDA-MB-231 cells, its effect on cell cycle regulatory proteins (Cyclin D1 and p21) were also checked. Cyclin D1 interacts with CDK4 and gets transported to the nucleus. Cyclin D1-CDK4 (cyclin-dependent kinase 4) complex induces phosphorylation of pRb (retinoblastoma protein) and inactivates it. E2F then dissociates from E2F/DP1/RB complex and induces the transcription of various genes involved in cell proliferation and genome maintenance^49^. However, Cyclin D1 independent of CDK4/6 can influence the activity of various transcription factors^50^. Cyclin D1 is also considered as a master regulator of G1-S phase transition during cell cycle^51^. The cyclin-dependent kinase inhibitor, p21 stimulates cell cycle arrest by inactivating cyclin-CDK4/6 complexes^49^. Despite its role as anti-proliferative, under certain conditions p21 can also function as an oncogene^52^. We observed significant decrease in Cyclin D1 and increase in p21Cip1/Waf1 levels respectively in miR-941 inhibitor transfected MDA-MB-231 cells. The decreased Cyclin D1 levels can also be due to PP2B activation by miR-941 inhibitor.

MiRNAs play an important role in the migration and invasion of cancer cells. Ectopic expression of miR-205 decreased the invasion and metastasis of MDA-MB-231 and prostate cancer (PC-3) cells^53^. Epithelial-mesenchymal transition (EMT) plays an important role in cancer progression and metastasis^54^. The role of E-cadherin in EMT and metastasis is well demonstrated in various tumours^55,56^. In cancer, matrix metalloproteinases (MMPs) are involved in proliferation, invasion, migration, differentiation, and angiogenesis by degrading extracellular matrix (ECM) components^57,58^. MMP-13, a collagenolytic MMP is over expressed in many types of invasive tumours, including breast carcinomas^59^. E-cadherin is a substrate for stromelysins (MMP-3) and matrilysins (MMP-7)^60^. Wound healing and transwell migration assays revealed that miR-941 inhibitor decreased the migration of MDA-MB-231 cells in comparison to negative control. MiR-941 inhibitor increased the expression of E-cadherin (Epithelial cell marker) and decreased the expression of MMP-13 (tumour marker for invasive breast cancer) respectively in MDA-MB-231 cells, which further confirms the role of miR-941 in migration and invasion of MDA-MB-231 cells.

As miRNA-941 inhibitor altered the protein expression of various cell cycle regulators like Cyclin D1, p21, and PP2B-B1, changes in other mitotic markers were checked out. Histone modifications are involved in the progression and arrest of cell cycle. In general, phosphorylation of histone H3 is associated with chromosome condensation prior to mitosis^61–63^. Phosphorylation of histone H3 at Ser10 and Ser28 appears in the G2/M phase, and both of these are the characteristic cell cycle markers to index the G2/M stages^62,64^. Interestingly, miR-941 inhibitor significantly dephosphorylated histone H3 at Ser10, suggesting that miR-941 inhibitor might have prevented chromosome condensation and also the entry of cells into mitosis. Phosphorylation and dephosphorylation of histone H3 at Ser10 are regulated by mitogen- and stress-activated kinase 1 (MSK1) and mitogen-activated protein kinase phosphatase 1 (MKP1) respectively^65,66^. A balance between protein phosphorylation by kinases and phosphatases determine the cell fate towards proliferation, differentiation or even death^67^. In order to examine their alterations, the protein expressions of p-MSK-1 and MKP-1 were checked. We observed decreased protein expression of p-MSK-1 in miR-941 inhibitor transfected MDA-MB-231 cells. We did not observe any change in the expression of MKP-1 with miR-941 inhibitor. This suggests that decreased p-MSK-1 might be also responsible for decreased phosphorylation of histone H3 at Ser 10. Okadaic acid is well known to increase the phosphorylation of histone H3 at Ser 10^68^. Interestingly, okadaic acid treatment alone increased the levels of hsa-miR-941 in MDA-MB-231 cells, suggesting that miRNA-941 may promote the proliferation of MDA-MB-231 cells by increasing phosphorylation of histone H3 at ser 10 residue. Further studies are required in detail to warrant any conclusion.

Fluorouracil (5-FU) is used as a single palliative treatment in combination with other antineoplastic agents for the therapy of breast cancers^69^. According to National Comprehensive Cancer Network guidelines, 5-fluorouracil, epirubicin and cyclophosphamide (FEC) adjuvant chemotherapy regimen is the recommended regimen for breast cancer^70^. Classical CMF (cyclophosphamide, methotrexate and 5-fluorouracil) has shown good clinical efficacy for the treatment of TNBC^71^. 30% of TNBC patients show pathological complete response (pCR) with standard anthracycline-, cyclophosphamide-, taxane-, and/or fluorouracil-based neoadjuvant chemotherapy^72^. Capecitabine, a prodrug of 5-FU is majorly used in the first line metastatic setting of TNBC due to its ease of administration and tolerability when compared to other anti-cancer drugs^73^. However, 5-FU resistance to cancer cells still remain as a major clinical concern^41^. Recently, Zhang et al. demonstrated that miR-587 decreased the sensitivity of 5-FU in colorectal cancer cells by downregulating serine/threonine protein phosphatase 2A regulatory subunit 1B (PPP2R1B) gene^74^. MiR-199a-3p improved the sensitivity of doxorubicin in hepatic cancer cells by down regulatingmTOR and c-Met^75^. Since 5-FU is widely used in TNBC and various miRNAs can modulate the sensitivity of chemotherapeutic agents in cancer cells^38,39^, we investigated whether miRNA-941 inhibitor increases the sensitivity of 5-FU at a dose which is less effective when given alone in MDA-MB-231 cells. Interestingly, it was observed that miRNA-941 inhibitor significantly increased the sensitivity of 5-FU in MDA-MB-231 cells when compared with 5-fluorouracil and MiR-941 inhibitor alone groups. Overexpression of thymidylate synthetase, Bcl-2, BCL-XL and Mcl-1 proteins are majorly responsible for 5-FU resistance in various cancers^41,76^. Apart from *TYMS* genetic polymorphisms, various miRNAs play a crucial role in altering sensitivity of cancer cells to 5-FU. MiR-192/215 improved the sensitivity of colorectal cancer cells to 5-FU by targeting cell cycle proteins such as p53, p21 and p27^77^. Induction of the protein levels of TYMS is observed after exposure to 5FU^78^. Thymidylate, required for DNA synthesis of the cells plays a crucial role for the survival of cancer cells. In our study, 5-FU induced inhibition of enzymatic activity of TYMS might be responsible for the induction of TYMS protein levels as a compensatory feedback mechanism. We observed that miR-941 inhibitor significantly decreased the protein expression of thymidylate synthetase in MDA-MB-231 cells. We did not observe decreased expression of thymidylate synthetase after treatment with 5-FU in MiRNA-941 inhibitor pretreated MDA-MB-231 cells, suggesting that alterations in cell cycle proteins such as p21 and CyclinD1 by MiR-941 inhibitor might be responsible for improved sensitization of 5-FU in MDA-MB-231 cells.To date, there are no reports available in literature showing the interaction of miRNA-941 with other anticancer agents. We report for the first time in our study that inhibition of miR-941 improves sensitization of MDA-MB-231 cells to 5-FU. Since MiRNA-941 acts as an oncogene in MDA-MB-231 cells, we speculate that inhibition of MiR-941 in MiR-941 over expressed cells may enhance sensitization of other anticancer drugs also apart from 5-FU. Further studies are required to warrant any conclusion. Moreover, recent report demonstrates that microRNA-941 protects endometrial cells from oxygen and glucose deprivation-re-oxygenation by activation of Nrf2 signalling through targeting Keap1, a Nrf2 suppressor protein^79^. Nrf2 overexpression promotes cancer progression and drug resistance in various cancers by activating several oncogenes such as matrix metallopeptidase 9, B-cell lymphoma 2, tumour necrosis factor α, and vascular endothelial growth factor A^80–82^. Nrf2 inhibitors act as chemosensitizers for the treatment of various cancers^83,84^. Nrf2-specific siRNA increased the sensitivity of cisplatin, gemcitabine, fluorouracil and camptothecin in pancreatic cancer cells^85,86^. Based on all these evidences, we speculate that inhibition of miR-941 might be also beneficial in increasing the chemosensitivity of various anti-cancer drugs to cancer cells by decreasing the activation of Nrf2 signalling. Future studies are required in detail to warrant any conclusion.

In support of in-vitro studies, breast cancer (MDA-MB-231) xenograft model of nude mice observations revealed that miR-941 inhibitor alone significantly suppressed tumour volume, further supporting our observations carried out in in vitro. MiR-941 inhibitor sensitized the tumour cells more to 5-FU and significantly suppressed tumour volume in comparison with all other groups, further supporting our observations carried out in in vitro. Body weight loss observed was minimal with combination group of miR-941 inhibitor and 5-FU. Highest extent of cytotoxic damage was observed in tumour of combination group of MiR-941 inhibitor and 5-FU treated animals. Previous studies have shown that increased interstitial space is responsible for increased penetration of paclitaxel and doxorubicin and apoptosis in head and neck cancers, and prostate cancer respectively^87,88^. In our study, the enhanced sensitivity of 5FU in miR-941 inhibitor + 5-fluorouracil group might be also probably due to increased penetration of 5FU to the tumours due to increased interstitial spaces by miR-941 inhibitor. A cartoon summarizing the findings of our study is shown in Supplementary Figure S6. Modulating the levels of miR-941 can be of profound clinical significance, as it provides novel therapeutic approach for breast cancer including triple negative breast cancer. However, further studies are required to extrapolate its role in other types of cancers overexpressing miRNA-941.

## Supplementary information

Supplementary Information.
