# Supplementary material for: MicroRNA-941 regulates the proliferation of breast cancer cells by altering histone H3 Ser 10 phosphorylation
Source: Sci Rep. 2020 Oct 21;10:17954. doi: 10.1038/s41598-020-74847-7 (PMC7578795; doi:10.1038/s41598-020-74847-7)

MicroRNA-941 regulates the proliferation of breast cancer cells by altering histone H3 Ser 10 phosphorylation.

Sunil Kumar Surapaneni, Zahid Rafiq Bhat, Kulbhushan Tikoo*

Laboratory of Epigenetics and Diseases, Department of Pharmacology and Toxicology,

National Institute of Pharmaceutical Education and Research (NIPER) S.A.S. Nagar, India

*Corresponding author: Prof. Kulbhushan Tikoo Department of Pharmacology and Toxicology,

National Institute of Pharmaceutical Education and Research (NIPER) S.A.S. Nagar, India

, Tel.9779199966; Fax: +91-172-2214692 Email: tikoo.k@gmail.com

Supplementary File

Supplementary Figure S1: Affymetrix Human miRNA profiling workflow

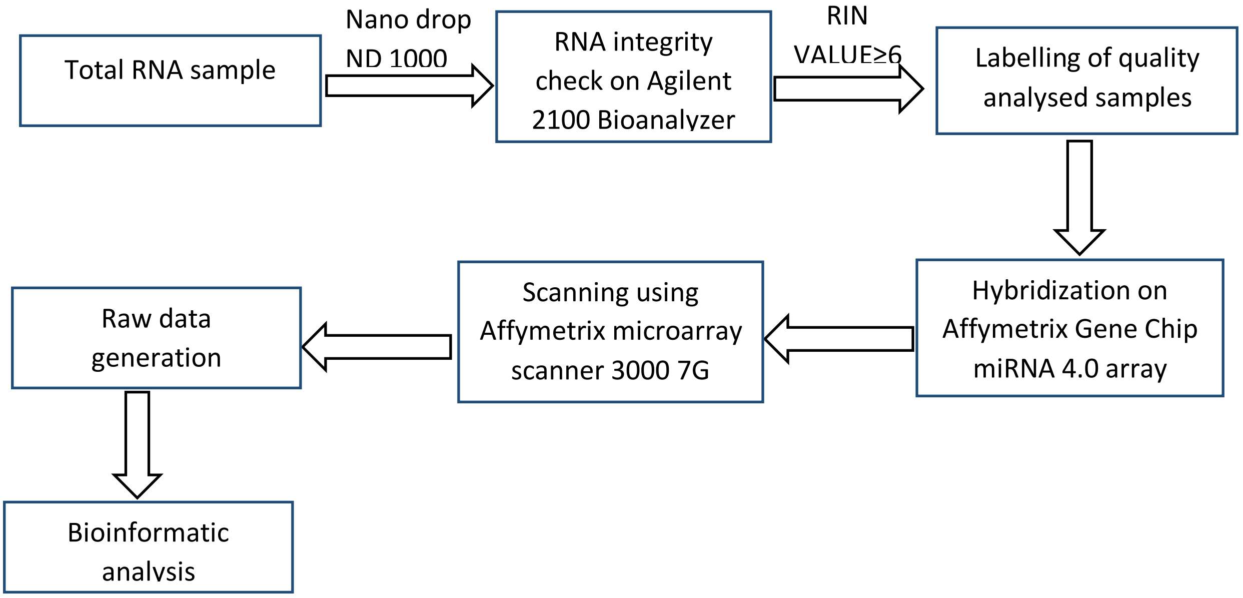

Hybridization conditions: 48°C and 60 rpm for 16-18 h

Supplementary Figure S2:

Bioinformatic Analysis

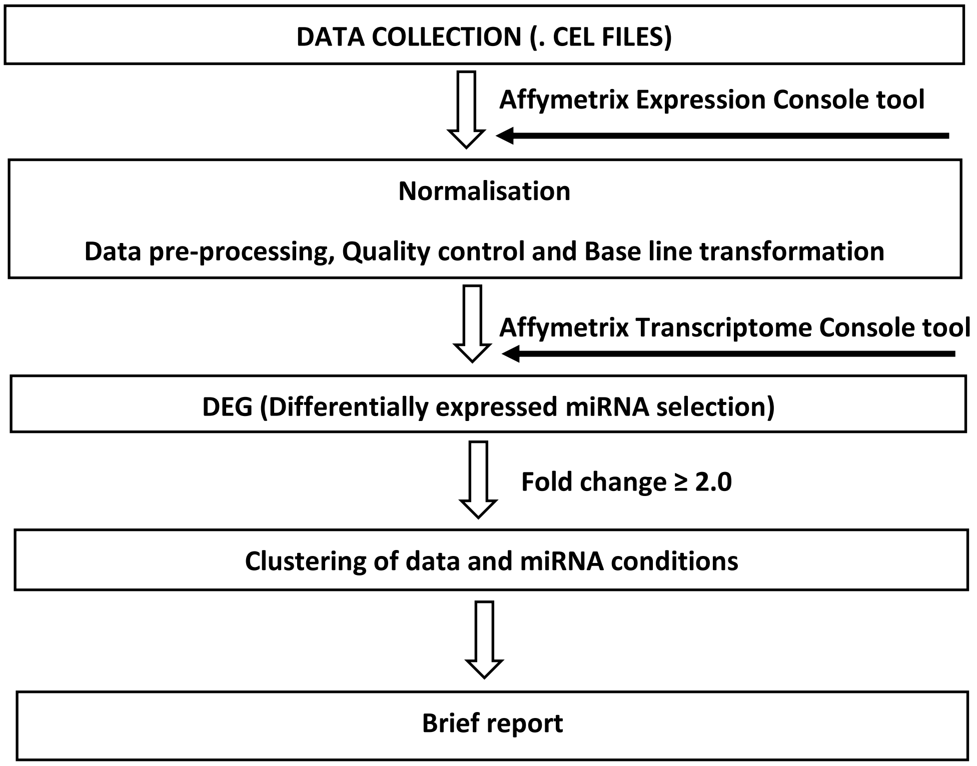

Supplementary Figure S3: Scatter plot of differentially expressed miRNAs

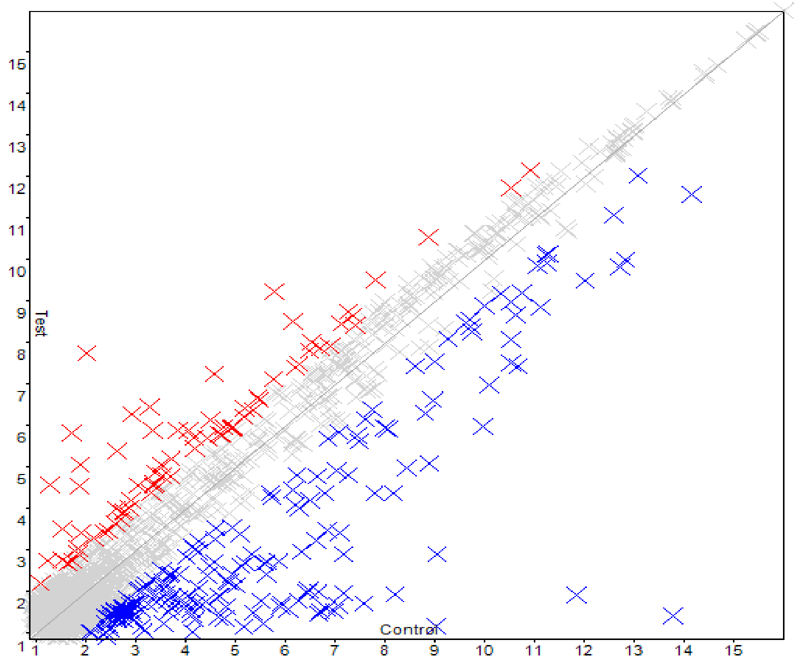

In the above figure, red colour shows up regulation of miRNAs & blue colour shows down regulation of miRNAs in TNBC cell line (MDA-MB 231) when compared to MCF10A cell line

Supplementary Figure S4: Hierarchical clustering images of upregulated (S4A-up) and downregulated (S4B-down) miRNAs respectively

Figure S4 shows heat maps of upregulated and downregulated miRNAs in MDA-MB-231 cell line when compared to MCF-10A cell line. These were represented as upregulated (S4A-up) and downregulated (S4B-down).

S4A-up

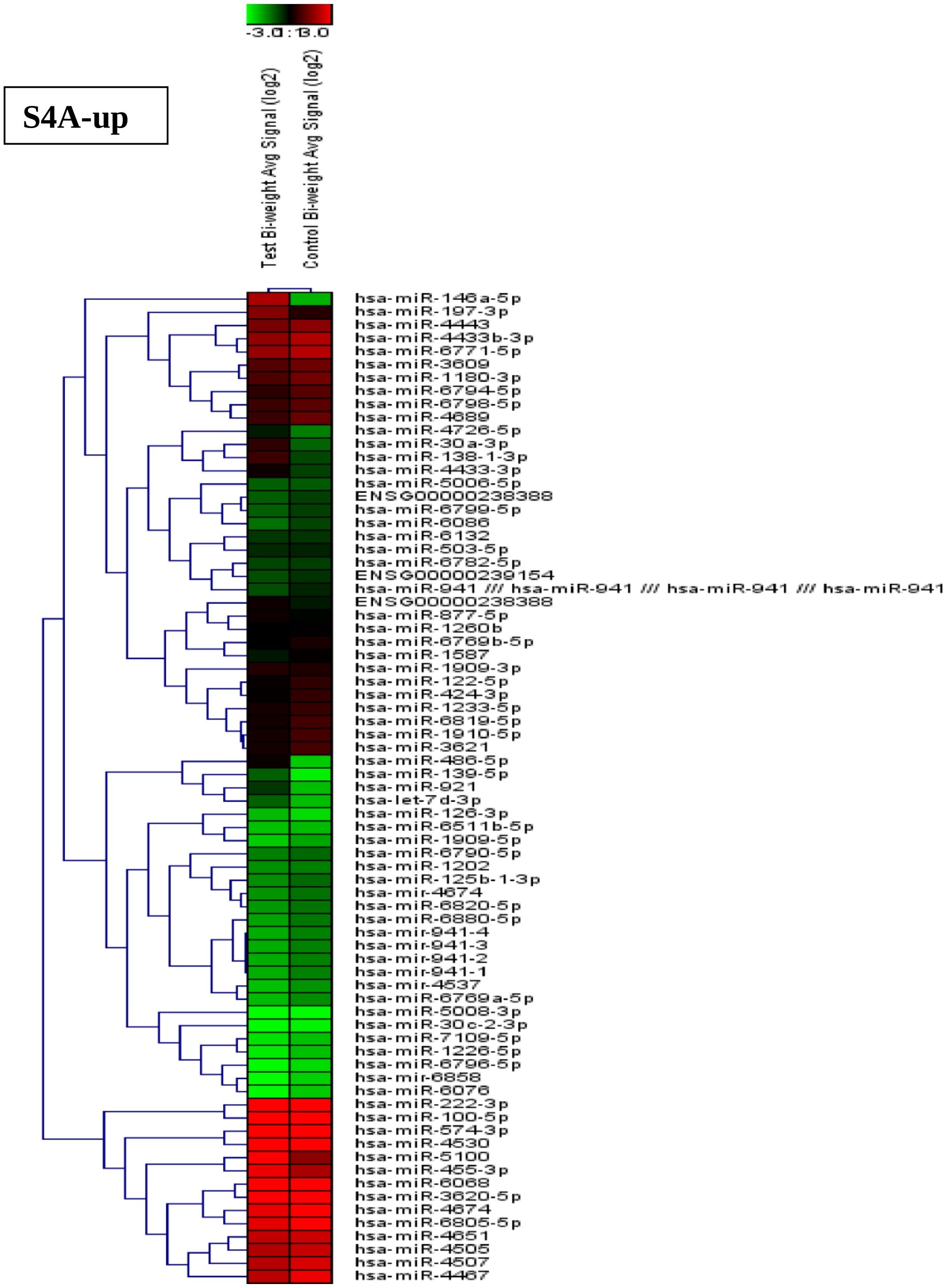

S4B-down

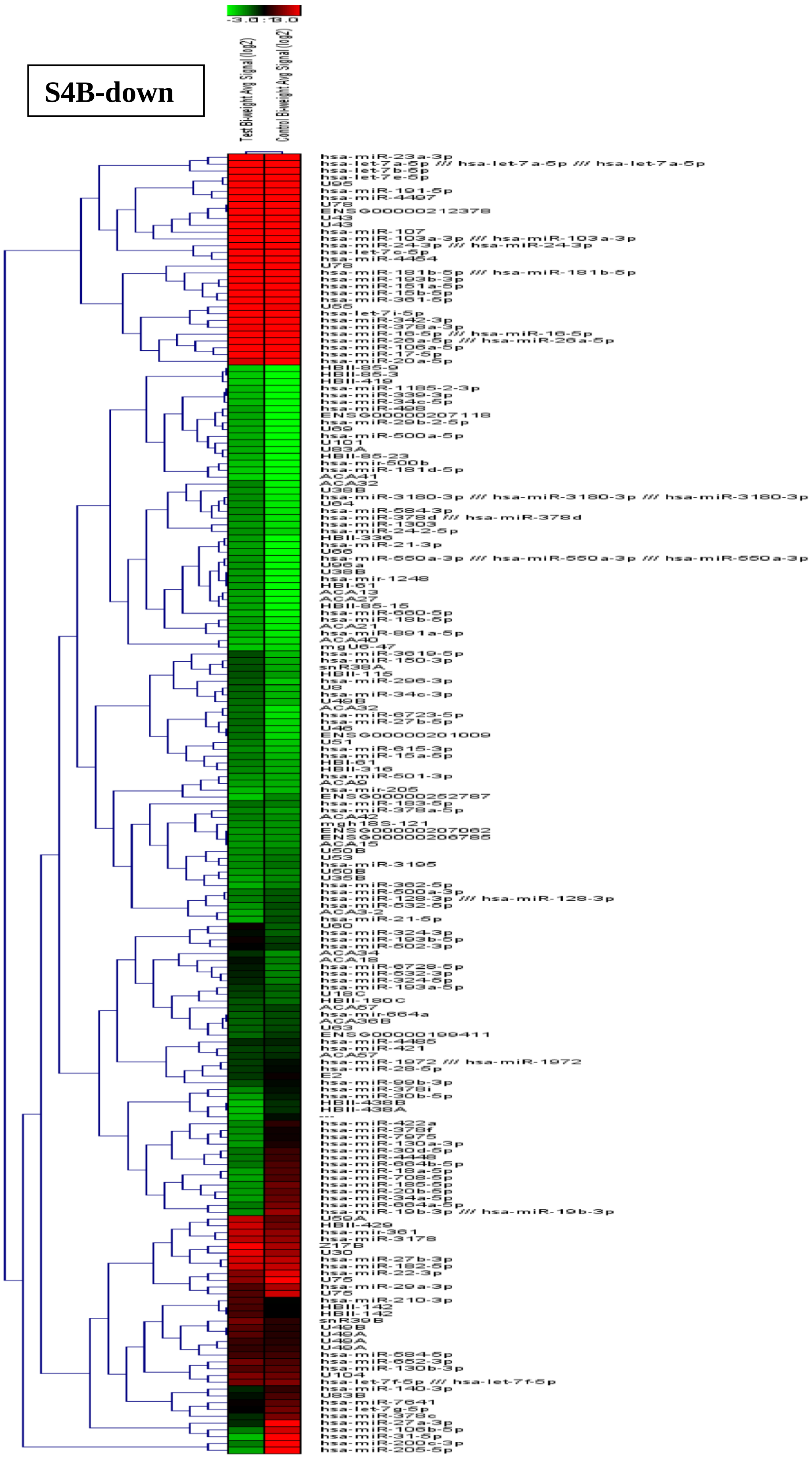
-3.0 : 13.0
Test Bi-weight Avg Signal (log2)
Control Bi-weight Avg Signal (log2)
hsa-miR-23a-3p
hsa-let-7a-5p /// hsa-let-7a-5p /// hsa-let-7a-5p
hsa-let-7b-5p
hsa-let-7e-5p
U95
hsa-miR-191-5p
hsa-miR-4497
U78
ENSG00000212378
U43
U43
hsa-miR-107
hsa-miR-103a-3p /// hsa-miR-103a-3p
hsa-miR-24-3p /// hsa-miR-24-3p
hsa-let-7c-5p
hsa-miR-4454
U78
hsa-miR-181b-5p /// hsa-miR-181b-5p
hsa-miR-193b-3p
hsa-miR-151a-5p
hsa-miR-15b-5p
hsa-miR-361-5p
U55
hsa-let-7i-5p
hsa-miR-342-3p
hsa-miR-378a-3p
hsa-miR-16-5p /// hsa-miR-16-5p
hsa-miR-26a-5p /// hsa-miR-26a-5p
hsa-miR-106a-5p
hsa-miR-17-5p
hsa-miR-20a-5p
HBII-85-9
HBII-85-3
HBII-419
hsa-miR-1185-2-3p
hsa-miR-339-3p
hsa-miR-34c-5p
hsa-miR-498
ENSG00000207118
hsa-miR-29b-2-5p
U69
hsa-miR-500a-5p
U101
U83A
HBII-85-23
hsa-mir-500b
hsa-miR-181d-5p
ACA41
ACA32
U38B
hsa-miR-3180-3p /// hsa-miR-3180-3p /// hsa-miR-3180-3p
U64
hsa-miR-584-3p
hsa-miR-378d /// hsa-miR-378d
hsa-miR-1303
hsa-miR-242-5p
HBII-336
hsa-miR-21-3p
U66
hsa-miR-550a-3p /// hsa-miR-550a-3p /// hsa-miR-550a-3p
U96a
U38B
hsa-mir-1248
HBI-61
ACA13
ACA27
HBII-85-15
hsa-miR-660-5p
hsa-miR-18b-5p
ACA21
hsa-miR-891a-5p
ACA40
mgU6-47
hsa-miR-3619-5p
hsa-miR-150-3p
snR38A
HBII-115
hsa-miR-296-3p
U8
hsa-miR-34c-3p
U49B
ACA32
hsa-miR-6723-5p
hsa-miR-27b-5p
U46
ENSG00000201009
U51
hsa-miR-615-3p
hsa-miR-15a-5p
HBI-61
HBII-316
hsa-miR-501-3p
ACA9
hsa-mir-205
ENSG00000252787
hsa-miR-183-5p
hsa-miR-378a-5p
ACA42
mgh18S-121
ENSG00000207062
ENSG00000206785
ACA15
U50B
U53
hsa-miR-3195
U50B
U35B
hsa-miR-362-5p
hsa-miR-500a-3p
hsa-miR-128-3p /// hsa-miR-128-3p
hsa-miR-532-5p
ACA3-2
hsa-miR-21-5p
U60
hsa-miR-324-3p
hsa-miR-193b-5p
hsa-miR-502-3p
ACA34
ACA18
hsa-miR-6728-5p
hsa-miR-532-3p
hsa-miR-324-5p
hsa-miR-193a-5p
U18C
HBII-180C
ACA57
hsa-mir-664a
ACA36B
U63
ENSG00000199411
hsa-miR-4485
hsa-miR-421
ACA57
hsa-miR-1972 /// hsa-miR-1972
hsa-miR-28-5p
E2
hsa-miR-99b-3p
hsa-miR-378i
hsa-miR-30b-5p
HBII-438B
HBII-438A
---
hsa-miR-422a
hsa-miR-378f
hsa-miR-7975
hsa-miR-130a-3p
hsa-miR-30d-5p
hsa-miR-4448
hsa-miR-664b-5p
hsa-miR-18a-5p
hsa-miR-708-5p
hsa-miR-185-5p
hsa-miR-20b-5p
hsa-miR-34a-5p
hsa-miR-664a-5p
hsa-miR-19b-3p /// hsa-miR-19b-3p
U59A
HBII-429
hsa-mir-361
hsa-miR-3178
Z17B
U30
hsa-miR-27b-3p
hsa-miR-182-5p
hsa-miR-22-3p
U75
hsa-miR-29a-3p
U75
hsa-miR-210-3p
HBII-142
HBII-142
snR39B
U49B
U49A
U49A
U49A
hsa-miR-584-5p
hsa-miR-652-3p
hsa-miR-130b-3p
U104
hsa-let-7f-5p /// hsa-let-7f-5p
hsa-miR-140-3p
U83B
hsa-miR-7641
hsa-let-7g-5p
hsa-miR-378c
hsa-miR-27a-3p
hsa-miR-106b-5p
hsa-miR-31-5p
hsa-miR-200c-3p
hsa-miR-205-5p

Supplementary Figure S5: Quantitative RT-PCR of down regulated miRNAs, which are observed in different pattern in comparison to miRNA microarray

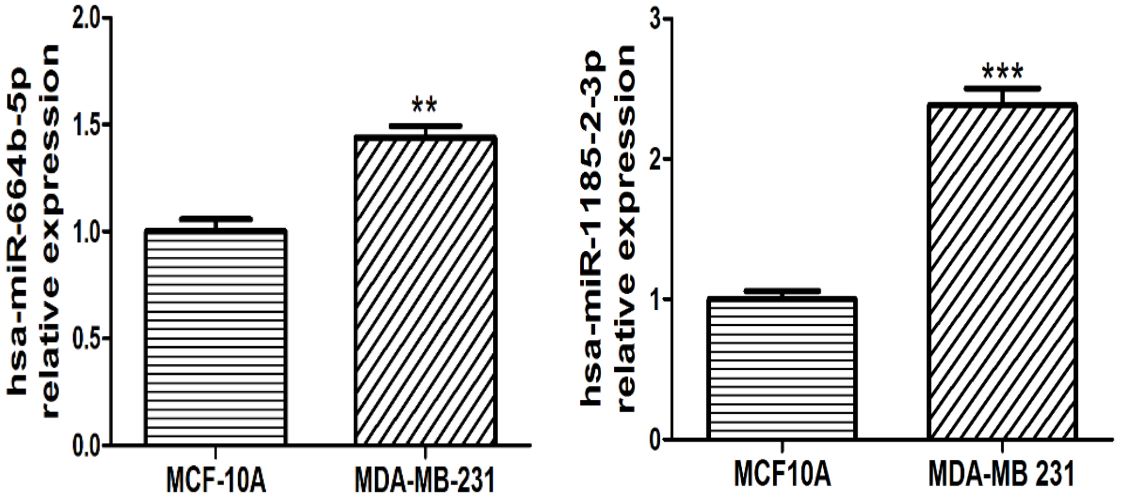

Supplementary Figure S5: Quantitative real-time PCR of hsa-miR-664b-5p and hsa-miR-1185-2-3p in MDA-MB-231 cell line when compared to MCF-10A cell line. Results shown were representative of three different experiments. All values expressed as mean ± S.E.M. ***p*<0.01 ****p*<0. 001 significant *vs* MCF-10A.

Supplementary Figure S6: Graphical abstract

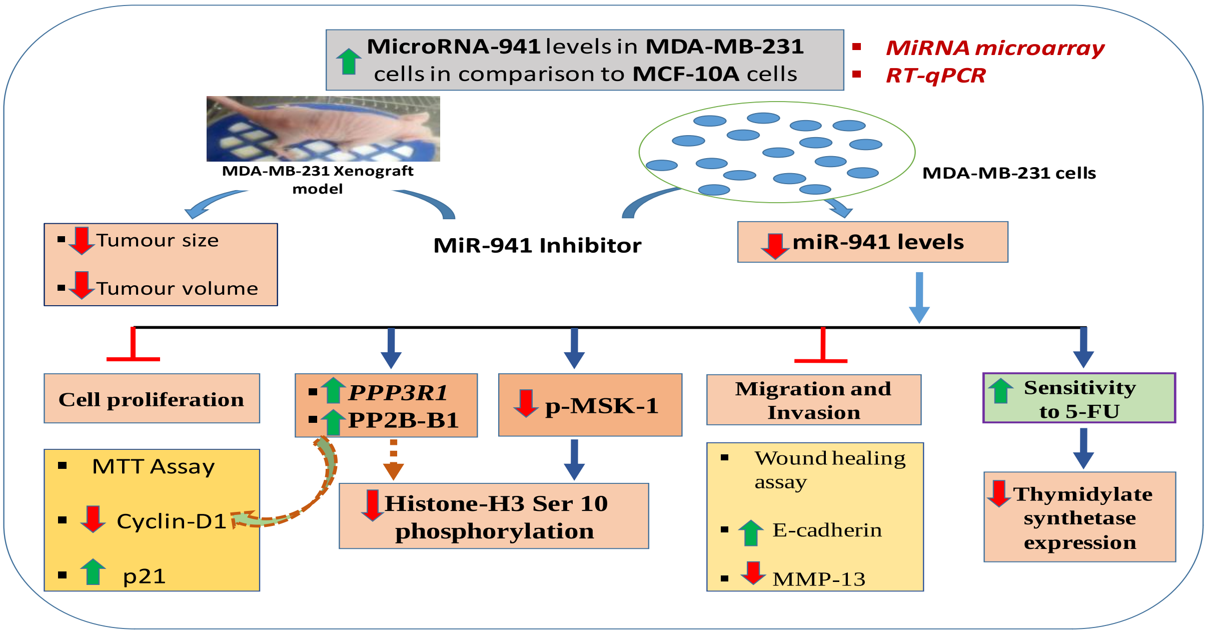

Supplementary Table S1: Sequences of primers used for mRNA quantification by RT-PCR

S.No.	***Gene name***	***Primer sequences***
1	***PPP3R1***	Forward: 5'-TGTGCTCACACTTTGATGCG-3'<br>Reverse: 5'-TGAGAGACGCCCTCAATGAA-3'
2	***GAB1***	Forward primer: 5'-GCCATTAACTGTGCTTCCCA-3'<br>Reverse primer: 5'-AGTGGACCAAAGTGCTGACT-3'
3	***RNF19A***	Forward primer: 5'-GGAGGAGGAGTTCGGGAAAG-3'<br>Reverse primer: 5'-CTTTAACTCCTCAGAGCGGC-3'
4	***NDE1***	Forward primer: 5'-AGTTTAAGGAGGCCTTCCCC-3'<br>Reverse primer: 5'-GTTCAAATCGCCTTCCCTCC-3'
5	***18S rRNA***	Forward primer: 5'-GCAATTATTCCCCATGAACG-3,<br>Reverse primer: 5'-AGGGCCTCACTAAACCATCC-3'

Supplementary Table S5: List of forward and reverse primers of *PPP3R1*, *NDE1*, *RNF19A* and *GAB1* genes used for mRNA quantification through quantitative RT-PCR.18S rRNA was used as reference gene.

Supplementary Table S2: Upregulated miRNAs in MDA-MB-231 cells in comparison to MCF-10A cell line

hsa-miR-455-3p	hsa-miR-4433-3p	hsa-miR-4651	hsa-miR-6771-5p
hsa-miR-486-5p	**hsa-miR-424-3p***	hsa-miR-6068	**hsa-miR-146a-5p***
hsa-miR-5100	hsa-miR-4467	hsa-miR-1202	hsa-miR-100-5p
hsa-miR-30a-3p	hsa-miR-126-3p	hsa-miR-4443	hsa-miR-1233-5p

hsa-miR-139-5p	hsa-miR-877-5p	hsa-miR-4674	hsa-miR-6799-5p
hsa-miR-921	hsa-miR-574-3p	hsa-miR-4505	hsa-miR-4674
hsa-miR-138-1-3p	hsa-miR-4530	hsa-miR-3620-5p	hsa-miR-4507
hsa-miR-4726-5p	**hsa-miR-1909-5p***	hsa-miR-6790-5p	**hsa-miR-941***
hsa-miR-7d-3p	hsa-miR-6132	hsa-miR-6769b-5p	hsa-miR-1909-3p
hsa-miR-197-3p	hsa-miR-1260b	hsa-miR-4433b-3p	hsa-miR-1180-3p
hsa-miR-6796-5p	hsa-miR-6511b-5p	hsa-miR-1587	hsa-miR-6820-5p
hsa-miR-4537	hsa-miR-5006-5p	**hsa-miR-222-3p***	hsa-miR-5008-3p
hsa-miR-6782-5p	**hsa-miR-30c-2-3p***	hsa-miR-122-5p	**hsa-miR-3609***
hsa-miR-6798-5p	hsa-miR-503-5p	hsa-miR-125b-1-3p	hsa-miR-1226-5p
hsa-miR-6076	hsa-miR-4674	hsa-miR-6880-5p	hsa-miR-941-4
hsa-miR-941-3	hsa-miR-941-2	hsa-miR-941-1	hsa-miR-6794-5p
hsa-miR-4689	hsa-miR-6769a-5p	hsa-miR-6858	hsa-miR-7109-5p
hsa-miR-1910-5p	hsa-miR-6819-5p	hsa-miR-3621	hsa-miR-6805-5p

Note: MiRNAs highlighted bold and with asterisk (*) symbol were selected for validation for our study

Supplementary Table S3: Downregulated miRNAs in MDA-MB-231 cells in comparison to MCF-10A cell line

hsa-miR-6723-5p	hsa-miR-532-3p	**hsa-miR-193b-5p***	hsa-miR-652-3p	**hsa-miR-24-3p***
hsa-miR-3619-5p	hsa-miR-500a-5p	hsa-miR-34c-3p	hsa-miR-107	hsa-miR-378a-3p
hsa-miR-498	hsa-miR-1248	hsa-miR-18-5p	hsa-miR-378a-5p	hsa-miR-664a
hsa-miR-29b-2-5p	hsa-miR-191-5p	hsa-miR-660-5p	hsa-miR-27b-3p	hsa-let-7b-5p
hsa-miR-23a-3p	hsa-miR-34c-5p	hsa-miR-7i-5p	hsa-miR-501-3p	hsa-miR-3195
hsa-miR-27b-5p	hsa-miR-3180-3p	hsa-miR-361-5p	hsa-miR-182-5p	hsa-miR-500a-3p
hsa-miR-6728-5p	hsa-miR-150-3p	hsa-miR-7a-5p	hsa-miR-183-5p	hsa-miR-128-3p
hsa-miR-21-3p	hsa-miR-324-5p	hsa-miR-1303	hsa-miR-103a-3p	hsa-let-7c-5p

hsa-miR-296-3p	hsa-miR-584-3p	hsa-miR-24-2-5p	hsa-miR-584-5p	hsa-miR-362-5p
hsa-miR-7e-5p	hsa-miR-324-3p	**hsa-miR-200c-3p***	hsa-miR-205	**hsa-miR-18a-5p***
hsa-miR-550a-3p	hsa-miR-210-3p	hsa-miR-891a-5p	hsa-miR-342-3p	hsa-miR-1972
hsa-miR-193b-3p	hsa-miR-15b-5p	hsa-miR-615-3p	hsa-miR-4485	hsa-miR-28-5p
hsa-miR-151a-5p	hsa-miR-4497	hsa-miR-361	hsa-miR-130b-3p	hsa-miR-106a-5p
hsa-miR-181b-5p	hsa-miR-500b	hsa-miR-502-3p	hsa-miR-26a-5p	hsa-miR-16-5p
**hsa-miR-1185-2-3p***	hsa-miR-378d	hsa-miR-193a-5p	hsa-let-7f-5p	hsa-miR-532-5p
hsa-miR-339-3p	hsa-miR-181d-5p	hsa-miR-3178	**hsa-miR-99b-3p***	hsa-miR-421
hsa-miR-17-5p	hsa-miR-30b-5p	hsa-miR-130a-3p	hsa-miR-19b-3p	hsa-let-7g-5p
hsa-miR-7641	hsa-miR-20a-5p	**hsa-miR-664b-5p***	hsa-miR-27a-3p	hsa-miR-4448
hsa-miR-140-3p	hsa-miR-378f	hsa-miR-15a-5p	hsa-miR-106b-5p	hsa-miR-185-5p
hsa-miR-29a-3p	hsa-miR-7975	hsa-miR-664a-5p	hsa-miR-31-5p	hsa-miR-34a-5p
hsa-miR-21-5p	hsa-miR-378c	hsa-miR-708-5p	hsa-miR-4454	hsa-miR-442a
hsa-miR-22-3p	hsa-miR-30d-5p	hsa-miR-20b-5p	hsa-miR-205-5p	hsa-miR-378i

Note: MiRNAs highlighted bold and with asterisk (*) symbol were selected for validation in our study

Supplementary table S4: Comparison of fold change values of dysregulated MiRNAs between miRNA microarray analysis and Quantitative RT-PCR analysis

S.NO	MiRNAs name	Fold change by miRNA microarray analysis	Fold change by Quantitative real-time PCR
1	hsa-miR-30c-2-3p	+ 2.85	+ 2.41
2	hsa-miR-424-3p	+ 2.06	+ 4.89
3	hsa-miR-941	+ 2.10	+ 9.74

4	hsa-miR-3609	+ 2.36	+ 2.44
5	hsa-miR-146a-5p	+ 3.85	+ 1.93
6	hsa-miR-1909-5p	+ 2.21	+ 1.68
7	hsa-miR-222-3p	+ 2.38	+ 4.30
8	hsa-miR-193b-5p	- 2.62	- 2.04
9	hsa-miR-18a-5p	- 4.37	- 1.72
10	hsa-miR-24-3p	- 5.64	- 2.38
11	hsa-miR-99b-3p	- 2.32	- 1.55
12	hsa-miR-200c-3p	- 6.12	- 2.88

Supplementary Table S5: Differentially expressed snoRNAs in MDA-MB-231 cells in comparison to MCF-10A cells

Upregulated snoRNAs (Unknown function)

ENSG00000238388
ENSG00000239154

Downregulated snoRNAs (H/ACA box)

ACA18	ACA32	ACA9	ACA15
U69	U64	ACA42	ACA36B
U66	ACA13	ACA40	E2
ACA34	ACA27	HBI-61	ACA3-2
HBI-61	ACA21	ACA41	ACA32

Downregulated snoRNAs (Unknown function)

ENSG00000207118
ENSG00000201009
ENSG00000212378
ENSG00000207062
ENSG00000206785
ENSG00000199411
ENSG00000252787

Downregulated snoRNAs (C/D box)

HBII-85-9	U55	U35B	U83B	U49A
HBII-85-3	U101	HBII-180C	U75	snR39B
HBII-336	U38B	HBII-316	U49A	U50B
U60	HBII-429	U49B	mgU6-47	U59A
U78	U83A	U18C	U104	HBII-142
HBII-419	snR38A	U43	U53	U49A
U46	U8	U51	U38B	U43
U95	HBII-85-23	U49B	mgh18S-121	HBII-115
U78	HBII-85-15	U30	U63	HBII-438B
U96a	Z17B	HBII-142	U75	HBII-438A
U50B				

Uncropped images of the blots

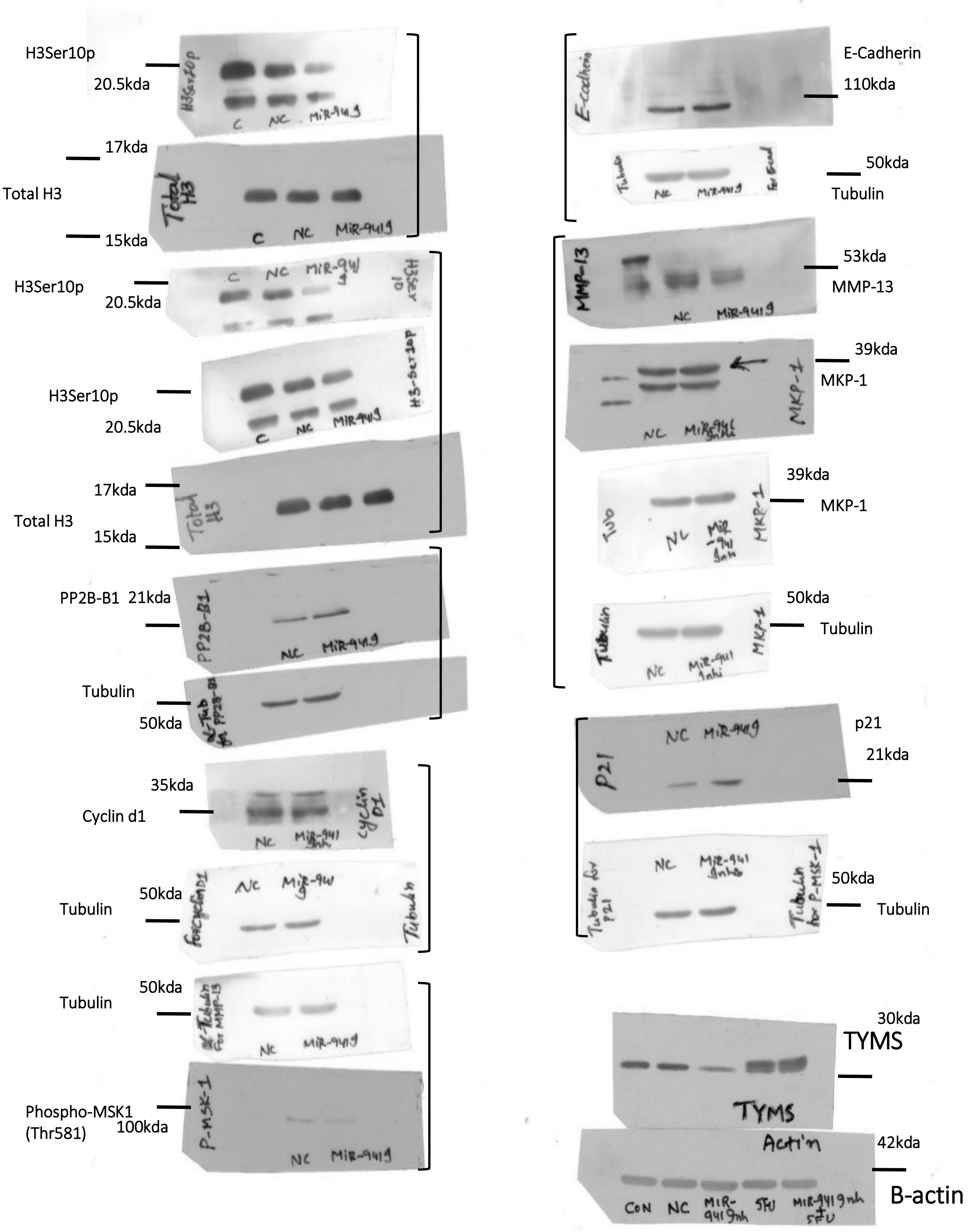

Supplement: Supplementary file 1 — Supplementary Information. [file 41598_2020_74847_MOESM1_ESM.pdf]
